# Integration of Phytomelatonin Signaling With Jasmonic Acid in Wound‐induced Adventitious Root Regeneration

**DOI:** 10.1002/advs.202413485

**Published:** 2025-01-23

**Authors:** Ying Liu, Xiaoyun Wang, Shirui Jing, Congyang Jia, Hongxin Li, Chonghua Li, Qiuyu He, Na Zhang, Yang‐Dong Guo

**Affiliations:** ^1^ College of Horticulture China Agricultural University Beijing 100193 China; ^2^ College of Plant Science and Technology Beijing University of Agriculture Beijing 102206 China

**Keywords:** adventitious root regeneration, phytomelatonin, phytomelatonin receptor, phytohormone crosstalk, signal transduction

## Abstract

Plants exhibit remarkable regenerative abilities under stress conditions like injury, herbivory, and damage from harsh weather, particularly through adventitious root formation. They have sophisticated molecular mechanisms to recognize and respond to wounding. Jasmonic acid (JA), a wound hormone, triggers auxin synthesis to stimulate root regeneration. Melatonin (MT), structurally similar to auxin, also significantly influences root induction, but its specific mechanism is unclear. Phytomelatonin's signal transduction is discovered in wound‐induced root formation, identifying SlPMTR1/2 as phytomelatonin receptors, transmitting signals to SHOOT BORNE ROOTLESS 1 (SlSBRL1), a key regulator of wound‐induced root regeneration, via the G protein α subunit 1 (SlGPA1). Additionally, SlPMTR1/2 is activated by JA, and targeted by SlMYC2. Overall, the specific mechanisms of phytomelatonin on wound‐induced root regeneration is uncovered and revealed a crosstalk between phytomelatonin and JA, offering new insights into plant repair mechanisms.

## Graphical abstract

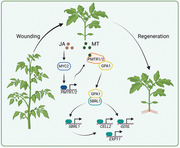

## 1 Introduction

Due to their fixed and immovable nature, plants often suffer mechanical damage from wind, rain, animal activity, human intervention, or equipment operation. Such abiotic stressors may cause serious damage to plant tissues and may also facilitate the invasion of pathogens. To cope with these challenges, plants have developed a robust regenerative ability, which is typified by the emergence of new shoots and/or roots following significant structural loss through a process termed de novo organogenesis.^[^ ^1^, ^2^, ^3^, ^4^, ^5^ ^]^

Adventitious roots (ARs), which originate from tissues other than the primary root system, are essential components of a plant's root architecture. They are critical for the plant's adaptive mechanisms in response to environmental changes. ARs can develop naturally or be artificially induced through wounding or the application of hormones. This feature enables plants to better cope with mechanical damage through regeneration, and AR is particularly valuable for large‐scale propagation of plants in horticultural and silvicultural practices.^[^ ^6^, ^7^, ^8^ ^]^

In the induction of AR triggered by wounding, a crucial event is the cell fate transition.^[^ ^9^ ^]^ In *Arabidopsis*, AR primordium initiation is primarily governed by the hormone auxin. This hormone serves as a master regulator in the early stages of ARs development. However, ARs formation modulation involves a complex hormonal interplay, with other plant hormones like JA, cytokinin, ethylene, gibberellin, abscisic acid, and phytomelatonin contributing to the overall efficiency of AR induction.^[^ ^6^, ^10^, ^11^ ^]^ The auxin signaling pathway can directly target root cell fate‐controlling genes, such as *WUSCHEL RELATED HOMEOBOX 11/12* (*WOX11/12*) and *LATERAL ORGAN BOUNDARIES DOMAIN 16* (*LBD16*).^[^ ^12^, ^13^ ^]^ SHOOT BORNE ROOTLESS1(SlSBRL1) is a recently discovered LBD transcription factor involved in ARs formation. The *slsbrl1* mutants could not produce ARs after excising the primary root, which highlights its crucial role in the regeneration of ARs after wounding.^[^ ^8^ ^]^

*AUXIN RESPONSE FACTOR 6/8* (*ARF6/8*) are positive regulators of ARs initiation, while *ARF17* is a negative regulator. They regulate the expression of three *auxin‐inducible Gretchen Hagen3* (*GH3*) genes, *GH3.3, GH3.5*, and *GH3.6*, encoding acyl‐acid‐amido synthetases. These three *GH3* genes are essential for fine‐tuning the ARs initiation in *Arabidopsis* hypocotyl by regulating JA homeostasis.^[^ ^14^ ^]^ The activation or repression of this progress is regulated by auxin/indole acetic acid repressors (Aux/IAAs) IAA6/9/17, TRANSPORT INHIBITOR1/AUXIN‐SIGNALLING F‐BOX (TIR1/AFB) TIR1 and AFB2.^[^ ^15^ ^]^ Wounding stimuli can activate ETHYLENE RESPONSE FACTOR 114/115 (ERF114/115), resulting in their heterodimerization with the SCARECROW‐LIKE 5/21 (SCL5/21) /PHYTOCHROME A SIGNAL TRANSDUCTION 1 (PAT1) transcription factors to activate the expression of *PHYTOSULFOKINE 2/55* (*PSK2/5*)*, WOUND INDUCED DEDIFFERENTIATION 1* (*WIND1*)*, ARF5*, and *DNA‐BINDING ONE FINGER 3.4* (*DOF3.4*), promoting cell proliferation, cellular reprogramming, stem cell identity, and periclinal cell division, respectively. DOF3.4 activates cell division through transcriptional activation of *CYCD3;3*.^[^ ^16^ ^]^ Among these signals, JA has been well characterized as the wound‐inducible hormone. Typically, JA is maintained at low concentrations but rapidly accumulates following wounding, activating the signaling pathway through key transduction factor SlMYC2.^[^ ^17^, ^18^, ^19^ ^]^ JA can activate the expression of *ERF115* and regulate ARs regeneration by promoting cytokinin biosynthesis. In addition, JA promotes the expression of the *auxin oxidation dioxygenase 1* (*DAO1*) gene, thereby regulating the feedback of crosstalk between auxin and JA.^[^ ^20^, ^21^ ^]^ In detached leaves, JA levels are highly induced, activating ERF109, which promotes the *ANTHRANILATE SYNTHASE α1* (*ASA1*) and upregulates auxin levels to stimulate AR induction. However, prolonged JA signaling detrimentally affects root organ development. The leaf explant partially shuts down JA signaling through the interaction between ERF109 and JASMONATE‐ZIM‐DOMAIN (JAZ) protein, thereby blocking ERF109 activity.^[^ ^22^ ^]^ Therefore, JA and auxin are involved in the complex regulatory network that controls the ARs regeneration of hypocotyls.

Due to the functional and structural similarities between phytomelatonin and other plant hormones/biomolecules, the crosstalk between phytomelatonin and other signaling molecules was often considered to regulate a wide range of plant growth and developmental processes. Exogenous application of melatonin promoted the induction of ARs in de‐rooted lupin hypocotyls, with melatonin's role as a root‐generation promoter resembling that of indole‐3‐acetic acid.^[^ ^10^ ^]^ This effect has also been observed in tomatoes.^[^ ^11^ ^]^ However, the molecular mechanism by which phytomelatonin regulates AR formation is still unclear.

Melatonin was considered an animal‐specific hormone until it was found widely distributed in plants.^[^ ^23^, ^24^ ^]^ The discovery of its receptors suggests its role as a plant hormone.^[^ ^25^, ^26^ ^]^ Exogenous application of melatonin has a significant impact on plant morphogenesis,^[^ ^27^, ^28^ ^]^ growth,^[^ ^29^, ^30^, ^31^ ^]^ development,^[^ ^32^, ^33^, ^34^ ^]^ and so on.^[^ ^35^, ^36^, ^37^, ^38^, ^39^ ^]^ Its function varies with concentration: at low levels, it mainly acts as a hormonal signal, and at high levels, it may excessively scavenge reactive oxygen species (ROS) besides signaling, leading to side effects.^[^ ^40^ ^]^ The phytomelatonin receptor, AtPMTR1, discovered in *Arabidopsis*, specifically binds melatonin with saturation and interacts with the G‐protein alpha subunit 1 (GPA1) to mediate signal transduction.^[^ ^26^ ^]^ Homologs of AtPMTR1 in species such as maize, alfalfa, tobacco, and cassava also effectively detect phytomelatonin signals, playing a vital role in signal transduction.^[^ ^41^, ^42^, ^43^, ^44^, ^45^, ^46^ ^]^

The crosstalk between phytohormones has always been a key focus in the study of plant regulation. Phytomelatonin has been shown to regulate the expression of auxin transporters genes *PIN1/3/7* ^[^ ^47^ ^]^ and to induce the formation of ARs by affecting the expression of these *PIN* genes ^[^ ^11^, ^48^ ^]^ and *MdWOX11*.^[^ ^49^ ^]^ Analysis of rice RNA‐seq data showed that several auxin response genes, including five *OsAux/IAAs* (*OsIAA1/9/10/20*/*27*), four *OsGH3s*, one *OsARF*, and one *OsSAUR*, were significantly upregulated after melatonin treatment.^[^ ^27^ ^]^ However, at high concentrations, melatonin treatment decreased the biosynthesis of auxin and the expression of PINs proteins in *Arabidopsis* by significantly reducing the transcript levels of the corresponding genes (auxin biosynthesis genes: *YUC1/2/5/6, TAR2*; auxin transporter genes: *PIN1/3/7*).^[^ ^50^ ^]^ Phytomelatonin affects auxin signal transduction by affecting its biosynthesis, binding, and transport, and enhancing the expression of auxin genes.^[^ ^27^, ^28^ ^]^ In terms of JA, melatonin treatment in tomato fruit significantly improved the resistance to gray mold (caused by *Botrytis cinerea*) by increasing the content of methyl jasmonate (MeJA), upregulating the expression of *lipoxygenase* (*SlLoxD*), *allene oxide cyclase* (*SlAOC*) and p*roteinase inhibitor II* (*SlPI‐ II*) genes and decreasing the expression of *SlMYC2* and *SlJAZ1*, thus regulating the H_2_O_2_ level and JA signaling transduction pathway.^[^ ^51^ ^]^ Melatonin pretreatment of melon seedlings under copper stress inhibited the biosynthesis of linoleic acid and downregulated the expression of *LOX*, thus controlling the content of JA in roots and promoting the development of lateral roots.^[^ ^52^ ^]^ Application of melatonin could down‐regulate the production of JA induced by salt stress which benefited seedling recovery.^[^ ^53^ ^]^ However, it is still unclear whether the crosstalk between phytomelatonin and other phytohormones is achieved through its receptors PMTR.

In this study, we identified phytomelatonin's role in the signal transduction of wound‐induced ARs regeneration and revealed its interaction with JA. We discovered that SlPMTR1/2, the tomato homologs of *Arabidopsis* phytomelatonin receptor AtPMTR1, act as phytomelatonin receptors. Upon detecting phytomelatonin signals, SlPMTR1/2 engages with the G protein SlGPA1 to transmit these signals. SlGPA1 then interacts with SlSBRL1, a key regulatory factor for wound‐inducible ARs regeneration, activating downstream gene expression and promoting ARs regeneration. Additionally, SlPMTR1/2 is activated by JA via direct transcriptional targeting of SlMYC2. These interactions allow JA to regulate AR induction upon wounding, not only by stimulating auxin biosynthesis but also working synergistically with phytomelatonin, enhancing the molecular regulatory mechanism of ARs regeneration.

## 2 Results

### 2.1 Exogenous Melatonin Promotes Induction of Adventitious Roots upon Damage Occurs

**Figure 1:**
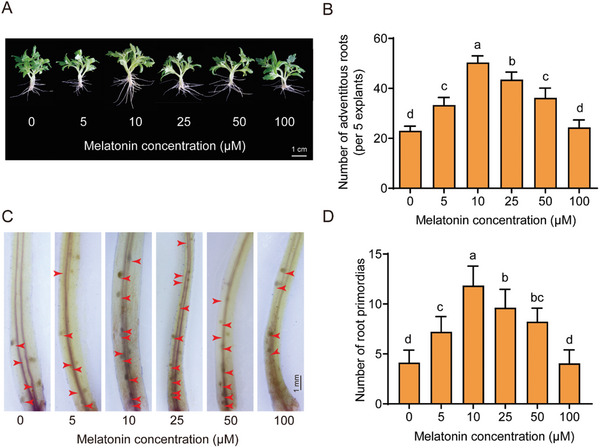
Melatonin enhances the regeneration of adventitious roots under mechanical injury. A) The primary roots were cut from 10‐day‐old germinated tomato hypocotyls and treated with 0, 5, 10, 25, 50, or 100 µm melatonin. Photographs were taken after 6 days of treatments. B) The number of ARs was recorded after 6 days of melatonin treatment. The data are presented as means ± SD from at least three independent experiments (n≥40 cuttings). Bars with different letters indicate significant differences (*p* < 0.05, one‐way ANOVA, Tukey's test). C) Root primordia were stained 4 days after removing the primary roots. D) The number of root primordia was recorded after 4 days of melatonin treatment. The data are presented as means ± SD from at least three independent experiments (n≥15 cuttings). Bars with different letters indicate significant differences (*p* < 0.05, one‐way ANOVA, Tukey's test). ARs, adventitious root; MT, melatonin.

Phytomelatonin facilitates the induction of ARs in wounded hypocotyls.^[^ ^10^, ^11^ ^]^ To confirm this, we examined AR phenotypes in wounded hypocotyls treated with 0—100 µm concentrations of exogenous melatonin, using 10‐day‐old tomato seedlings. After cutting the primary roots, we applied melatonin and observed AR development after a 6‐day period. Similar to auxin, melatonin's effect on AR formation was concentration‐dependent. Exogenous melatonin significantly increased AR numbers compared to untreated samples (*p *< 0.05), peaking at 10 µm before declining at higher concentrations (**Figure** **1**A,B). The process of AR formation starts with root primordia initiation. Four days after cutting the primary roots, we stained hypocotyl explants with Schiff's reagent, which showed that melatonin enhances root primordia formation in a concentration‐dependent manner, maximizing root primordia at 10 µm (Figure 1C,D). These results demonstrate the positive role of melatonin in the induction of ARs upon damage occurs.

### 2.2 Membrane Proteins SlPMTR1/2 are Phytomelatonin Receptors in Tomato

**Figure 2:**
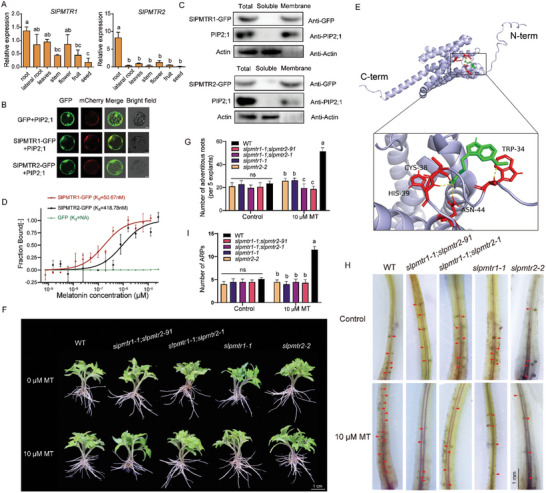
SlPMTR1/2 are tomato phytomelatonin receptors. A) The expression of *SlPMTR1/2* in different tomato plant tissues. The data are presented as means ± SD from at least three independent experiments. Bars with different letters indicate significant differences (*p* < 0.05, one‐way ANOVA, Tukey's test). B) Cellular localization of SlPMTR1/2 was observed using confocal microscopy in tomato mesophyll protoplasts 48 h post‐transformation. SlPMTR1/2‐GFP signals overlap with the plasma membrane marker PIP2;1‐mCherry. Bar = 25 µm. C) Immunoblotting confirmed GFP labeled SlPMTR1/2 in the membrane protein components of transgenic *Nicotiana benthamiana*. PIP2;1, membrane protein marker; Actin, soluble protein marker. D) Microscale thermophoresis (MST) analysis demonstrated the in vitro binding activity of SlPMTR1/2 protein to melatonin. MBP‐His‐SlPMTR1/2‐GFP and MBP‐His‐GFP protein were incubated with melatonin at varying concentrations for 30 min at 22 °C, and fluorescence data was analyzed using NTAnalysis software from three independent experiments. E) Prediction of SlPMTR1 and melatonin binding based on molecular docking. Melatonin (green) is covalently bound to four amino acids (Trp34, Cys38, His39, Asn44; red) on SlPMTR1. F) Primary roots from 10‐day‐old germinated tomato plants were cut and treated with or without 10 µm melatonin. Photographs were taken after 6 days of treatments. G) The number of ARs in (F) (n≥35 cuttings). H) Staining of root primordia on the fourth day after cutting the primary roots from hypocotyls. I) The Number of root primordia in (H) (n≥10 cuttings). Data in (G, I) are presented as means ± SD from at least three independent experiments, Bars with different letters indicate significant differences (*p* < 0.05, one‐way ANOVA, Tukey's test).

The identification of phytomelatonin receptors AtPMTR1 suggests phytomelatonin as a plant hormone,^[^ ^26^ ^]^ with further studies revealing their widespread presence across plant species.^[^ ^41^, ^43^, ^44^, ^46^ ^]^ By analyzing the tomato genome using the AtPMTR1 protein sequence, we found two highly similar genes *Solyc01g098210* and *Solyc06g069490*, naming them *SlPMTR1* and *SlPMTR2*. SlPMTR1 and SlPMTR2 share 62.42% and 59.23% sequence identity with AtPMTR1, respectively (Figure S1A, Supporting Information). Using *Arabidopsis* AtPMTR1 as a query, 16 high homologs were identified in dicot and monocot plants by BLASTP analysis (http://www.ncbi.nlm.nih.gov/), including the confirmed phytomelatonin receptors ZmPMTR1^[^ ^41^ ^]^ and MePMTR1.^[^ ^46^ ^]^ Phylogenetic analysis indicates that SlPMTR1 is more related to AtPMTR1,^[^ ^26^ ^]^ while SlPMTR2 is closer to ZmPMTR1^[^ ^41^ ^]^ (Figure S1B, Supporting Information). RT‐qPCR experiments showed *SlPMTR1* expression is present in all tomato tissues, while *SlPMTR2* had the highest expression level in roots (**Figure** **2**A). Meanwhile, the expression levels of *SlPMTR1* and *SlPMTR2* could also be triggered by melatonin treatment (Figure S1C, Supporting Information).

The bioinformatic analysis indicates that SlPMTR1/2 are seven‐transmembrane proteins with N‐term outside and C‐term inside the plasma membrane (Figure S1D, Supporting Information). We confirmed the subcellular localization of SlPMTR1/2 proteins by transiently overexpressing SlPMTR1/2‐GFP (green fluorescent protein) in tomato protoplasts. Under microscopy, SlPMTR1/2‐GFP fluorescence overlapped with PIP2;1‐mCherry, a marker for the plasma membrane,^[^ ^54^ ^]^ at the plasma membrane (Figure 2B). Immunoblotting assays showed that similar to the control membrane protein PIP2;1, only anti‐GFP signals antibodies were detected in the membrane fraction. No SlPMTR1/2‐GFP signal appeared in the soluble fraction (Figure 2C). These results confirm that SlPMTR1/2 are plasma membrane proteins.

To investigate whether SlPMTR1/2 are functional melatonin receptors in tomatoes, we expressed and purified 6×Histidine (His)‐maltose binding protein (MBP)‐GFP and 6×His‐MBP‐SlPMTR1/2‐GFP proteins in *E. coli*, and performed microscale thermophoresis (MST) experiment. The MST data showed that 6×His‐MBP‐SlPMTR1/2‐GFP strongly bind to melatonin, with K_d_ values of 50.67 nm for SlPMTR1 and 418.78 nm for SlPMTR2, while the negative control 6×His‐MBP‐GFP protein could not bind to melatonin (Figure 2D). This indicates that SlPMTR1/2 act as phytomelatonin receptor, with SlPMTR1 has a higher affinity for melatonin. Molecular docking identified the melatonin binding site on SlPMTR1, involving residues W34, C38, H39, and N44, primarily in the N‐terminal extracellular region (Figure 2E), supporting its role as a phytomelatonin receptor.

### 2.3 Phytomelatonin‐Induced AR in Wounded Tomato Hypocotyl is SlPMTR1/2 Dependent

To study the role of SlPMTR1/2 in the phytomelatonin signaling pathway, *slmptr1/2* mutants were generated. Four independent homozygous gene‐edited lines from T_2_ progeny were identified: two single mutants, *slpmtr1‐1* (1 bp deletion for *SlPMTR1*) and *slpmtr2‐2* (2 bp deletion for *SlPMTR2*), and two double mutants, *slpmtr1‐1*;*slpmtr2‐1* (1 bp deletion for *SlPMTR1* and 1 bp deletion for *SlPMTR2*) and *slpmtr1‐1*;*slpmtr2‐91* (1 bp deletion for *SlPMTR1* and 91 bp deletion for *SlPMTR2*), all of which cause premature coding termination. SlPMTR1 and SlPMTR2 both have a conserved transmembrane domain, and gene editing disrupts this conserved domain (Figure S2, Supporting Information).

At a concentration of 10 µm, melatonin treatment increased ARs in WT hypocotyls after primary root cutting, with no effect in various *slpmtrs* mutants (Figure 2F,G). Similarly, on the 4th day of treatment, melatonin significantly increased the number of root primordia in WT, but not in the various *slpmtrs* mutants (Figure 2H,I). This indicates that the induction of ARs by melatonin is impaired in various *slpmtrs* mutants, indicating that SlPMTR1/2 is essential for phytomelatonin signal perception in the induction of ARs in wounded tomato hypocotyl.

### 2.4 SlMYC2 Mediates Crosstalk between JA and Phytomelatonin by Regulating *SlPMTR1/2* Expression

JA, a hormone that rapidly accumulates in response to plant injury,^[^ ^17^ ^]^ activates signaling pathways through the key transcription factor SlMYC2 and cooperates with auxin to regulate the induction of ARs.^[^ ^55^ ^]^ The *slmyc2* mutant produced significantly fewer ARs than WT when the primary roots of seedlings were cut, confirming the participation of SlMYC2 in the induction of AR under mechanical injury (Figure S3A–C, Supporting Information).

**Figure 3:**
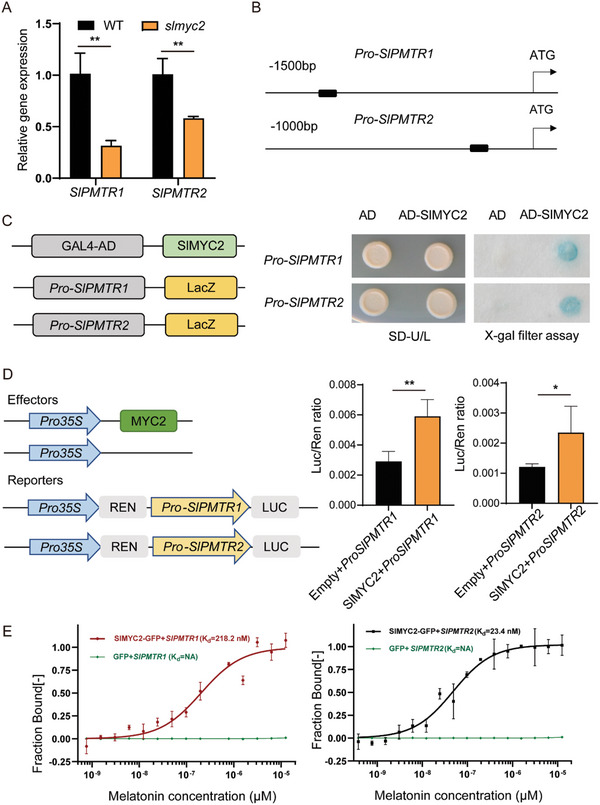
SlMYC2 directly activates the expression of *SlPMTR1/2*. A) The expression of *SlPMTR1/2* in WT and *slmyc2* mutant. Asterisks indicate a significant difference from WT according to Student's t‐test at **p  < 0.01. B) Schematic of the *SlPMTR1/2* promoter showing the G‐box (black box). C) Yeast one‐hybrid assays confirmed SlMYC2 binding to the promoter region of *SlPMTR1/2*. D) Dual‐luciferase reporter assays show that SlMYC2 activates the expression of *SlPMTR1/2*. The LUC/REN ratio represents the relative activity of the *SlPMTR1/2* promoter. Data are mean ± SD from five independent replicates, with significant differences from control at *p < 0.05 and **p < 0.01 (Student's t‐test). E) MST experiments verify the in vitro binding activity of SlMYC2 protein to *SlPMTR1/2* promoter fragments. MBP‐His‐SlMYC2‐GFP or MBP‐His‐GFP proteins were incubated with *SlPMTR1/2* promoter fragments in MST reaction buffer for 30 min at 22 °C, and thermophoresis signals were analyzed using NTAnalysis software from three independent experiments.

ChIP‐seq analysis by Du et al.^[^ ^56^ ^]^ indicates that *SlPMTR1/2* may be downstream of SlMYC2. In the *slmyc2* mutant, the expressions of *SlPMTR1* and *SlPMTR2* significantly decreased (**Figure** **3**A). These evidences prompt us to investigate whether SlMYC2 regulates the transcription of *SlPMTR1/2*. The G‐box motif has been widely reported as the SlMYC2 binding site.^[^ ^56^ ^]^ By analyzing the promoter sequence of *SlPMTR1/2*, we identified a G‐box (CACATG) located at position ‐1206 bp in *SlPMTR1's* promoter and ‐248 bp in *SlPMTR2's* (Figure 3B). Y1H assay showed that SlMYC2 specifically binds to G‐box motifs of *SlPMTR1* and *SlPMTR2* (Figure 3C). The relative LUC/REN ratios of the tobacco leaves co‐transformed with the SlMYC2 and *SlPMTR1/2* promoters were significantly higher than that of the leaves co‐transformed with the empty pGreenII 62‐SK vector and *SlPMTR1/2* promoters (Figure 3D). We expressed and purified 6×His‐MBP‐GFP and 6×His‐MBP‐SlMYC2‐GFP proteins in *E. coli*, and performed MST experiment. The MST data showed strong binding ability of 6×His‐MBP‐SlMYC2‐GFP to 60‐bp *SlPMTR1/2* promoter fragments containing the G‐box, with K_d_ values of 218.2 nm for *SlPMTR1* and 23.4 nm for *SlPMTR2*, while the negative control 6×His‐MBP‐GFP protein could not bind to *SlPMTR1/2* promoter fragments (Figure 3E). These results indicate that SlMYC2 positively regulates *SlPMTR1/2* transcription by directly binding to their promoters.

**Figure 4:**
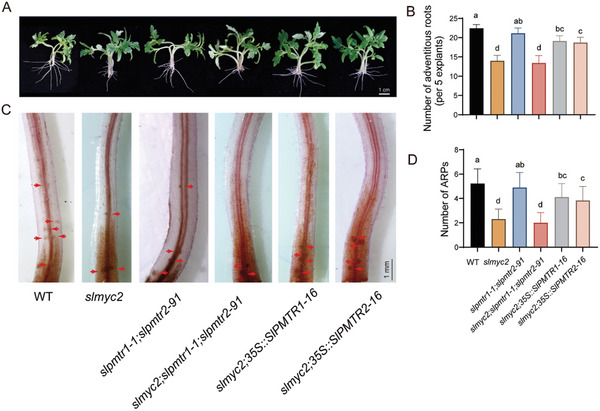
SlMYC2 and SlPMTR1/2 contribute to AR induction following mechanical injury. A) The primary roots of 10‐day‐old germinated tomato seedlings were removed, and photographs were taken after 6 days of treatment. B) AR numbers in various lines were assessed after 6 days, with data shown as means ± SD from at least three independent experiments (n≥35 cuttings) Different letters indicate significant differences (*p* < 0.05, one‐way ANOVA, Tukey's test). C) Staining of root primordia on the fourth day after cutting the primary roots from hypocotyls. D) The number of root primordia after 4 days of treatment. The data are presented as means ± SD from at least three independent experiments (n≥17 cuttings). Bars with different letters indicate significant differences (*p* < 0.05, one‐way ANOVA, Tukey's test).

SlMYC2 directly regulates *SlPMTR1/2*, suggesting that JA likely regulates ARs induction in coordination with phytomelatonin. We analyzed the expression of *SlMYC2* and *SlPMTR1/2* in the hypocotyls within 96 h after cutting the primary roots. As reported, *SlMYC2* rapidly responds to damage and peaks at 1 h post‐injury, then decreases and stabilizes. The response of both *SlPMTR1* and *SlPMTR2* was later than *SlMYC2*, with *SlPMTR1* peaking at 1, 9, and 48 h, while *SlPMTR2* peaks at 3 and 48 h (Figure S3D, Supporting Information). To investigate the role of SlMYC2 and SlPMTR1/2 in the induction of ARs under mechanical damage, we overexpressed *SlPMTR1* and *SlPMTR2* in *slmyc2* and generated a triple mutant of *slmyc2*;*slpmtr1‐1*;*slpmtr2‐91* (Figure S4A–C, Supporting Information). After 6 days post‐root cutting, ARs counts showed decreased ARs in *slmyc2* single and triple mutants compared to WT; the *slpmtr1‐1*;*slpmtr2‐91* mutant was not significantly changed compared to WT, but *slmyc2*;*35S::SlPMTR1* and *slmyc2*;*35S::SlPMTR2* restored ARs numbers in *slmyc2* (**Figure** **4**A,B). Similarly, the staining of root primordia showed the same pattern (Figure 4C,D). Similar results were observed when lateral branches were cut to induce the ARs in these materials (Figure S4D,E, Supporting Information). This indicates that activating melatonin signaling can partially offset impaired JA signaling, promoting AR regeneration.

### 2.5 SlPMTR1/2‐Regulated Gene Transcriptome Profiling during Phytomelatonin Signaling

We conducted RNA‐seq experiments to elucidate the phytomelatonin signal transduction in wound‐inducible ARs formation, comparing the transcriptome profiles of *slpmtr1‐1*;*slpmtr2‐91*, and wild‐type wounded hypocotyl with and without melatonin treatment. The total number of reads, mapping reads, and mapping read percentage in each replication are shown in Figure S5 (Supporting Information).

**Figure 5:**
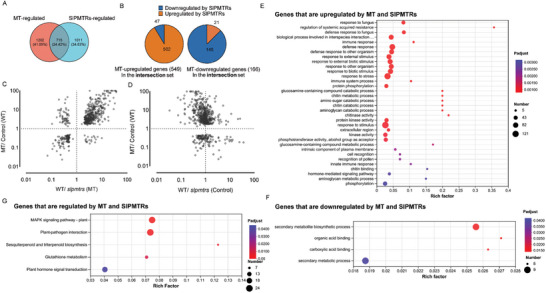
Gene set of receptor‐dependent MT‐regulated genes identified through pairwise RNA‐Seq comparisons. A) Venn diagrams of melatonin‐regulated genes (differentially expressed between melatonin‐treated and untreated wild‐type plants; FDR‐adjusted *p* value < 0.05) and SlPMTR1/2‐regulated genes (differentially expressed between melatonin‐treated *slpmtr1/2* and the wild type, FDR‐adjusted *p* value < 0.05). Overlapping regions highlight coregulated genes. Numbers of significantly differentially expressed genes (FDR‐adjusted *p* value < 0.05) are shown. B) Breakdown of SlPMTR1/2‐regulated genes among 715 genes coregulated by melatonin and SlPMTR1/2, showing up‐ and downregulated genes. C, D) Biplots of expression ratios from RNA‐seq. White circles represent 715 coregulated genes. Each point shows the average expression ratio of three biological replicates: (C) wild‐type melatonin‐treated vs untreated; (D) wild‐type vs *slpmtr1/2* with and without melatonin treatment. E, F) GO analysis of genes upregulated or downregulated by melatonin and SlPMTR1/2. Categories with *p* ≤ 0.05 were overrepresented among the co‐regulated genes. G) KEGG pathway analysis of genes regulated by melatonin and SlPMTR1/2. Categories with *p* ≤ 0.05 were overrepresented among the co‐regulated genes.

Pairwise RNA‐seq data comparisons identified 1917 genes with differential expression between melatonin‐treated and untreated wild‐type plants (FDR‐adjusted *p* value < 0.05) as melatonin‐regulated genes. Additionally, we identified 1726 genes with significant expression differences between melatonin‐treated *slpmtr1‐1*;*slpmtr2‐91*, and wild‐type seedlings as SlPMTR1/2‐regulated genes in our study on phytomelatonin signaling (**Figure** **5**A). Analyses of these genes revealed that 37.3% (715 of 1917) of the melatonin‐regulated genes were also governed by SlPMTR1/2 (Figure 5A), highlighting its pivotal role in melatonin‐regulated gene transcription.

Among the 715 genes coregulated by melatonin and SlPMTR1/2, 549 were upregulated and 166 were downregulated by melatonin. Interestingly, 91.4% (502 of 549) of melatonin‐upregulated genes were also upregulated by SlPMTR1/2, classified as differentially expressed genes with higher expression levels in melatonin‐treated wild type vs melatonin‐treated *slpmtr1‐1*;*slpmtr2‐91* plants (Figure 5B–D). Conversely, 87.3% (145 of 166) of melatonin‐downregulated genes were similarly affected by SlPMTR1/2, showing lower levels under the same conditions (Figure 5B–D). These results suggest that SlPMTR1/2 transduces phytomelatonin signals by enhancing melatonin‐induced and repressing melatonin‐repressed gene expression. GO analysis indicated that melatonin and SlPMTR1/2‐upregulated genes are enriched in defense response pathways, systemic acquired resistance, interspecific interactions, immune responses and stress responses (Figure 5E). Melatonin and SlPMTR1/2‐downregulated genes are enriched in pathways related to the secondary metabolite biosynthetic process, organic acid and carboxylic acid binding (Figure 5F). KEGG analysis highlights that genes co‐regulated by melatonin and SlPMTR1/2 are mainly involved in the MAPK cascade, plant‐pathogen interactions, hormone signal transduction, glutathione metabolism, amino sugar and nucleotide sugar metabolism (Figure 5G). Given that regeneration and defense are interconnected facets of the plant wound responses,^[^ ^57^ ^]^ the co‐regulation of melatonin and SlPMTR1/2 gene enrichment in defense and immunity has once again demonstrated its role in wound‐induced regeneration.

### 2.6 Phytomelatonin‐SlPMTR1/2 Signaling Promotes ARs Generation through Activating *SlSBRL1* Expression

**Figure 6:**
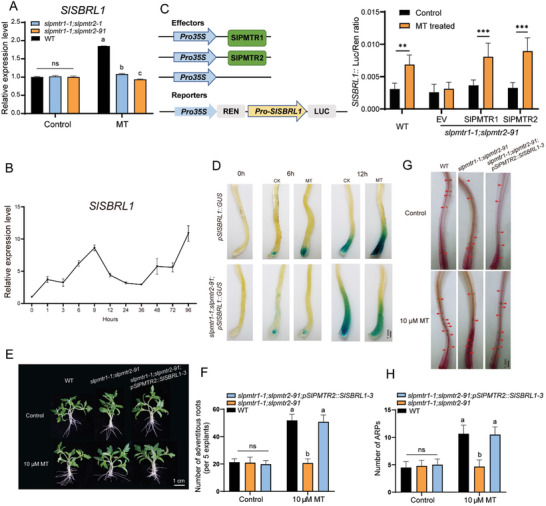
SlSBRL1 acts downstream of SlPMTR1/2 and contributes to melatonin‐induced AR formation under mechanical injury. A) The expression of *SlSBRL1* was analyzed in WT, *slpmtr1‐1*;*slpmtr2‐91*, and *slpmtr1‐1*;*slpmtr2‐1* with or without exogenous melatonin. The data are presented as means ± SD from at least three independent experiments. Different letters indicate significant differences (*p* < 0.05, one‐way ANOVA, Tukey's test). B) The temporal expression patterns of *SlSBRL1* in the wounded tomato seedling hypocotyls over 96 h. C) Melatonin promoted *SlSBRL1::LUC* expression in a SlPMTR1/2‐dependent manner. *slpmtr1‐1*;*slpmtr2‐91* protoplasts transfected with *35S::RLUC‐SlSBRL1::LUC*, along with empty vector (EV), SlPMTR1 or SlPMTR2, were treated with melatonin, and subjected to *SlSBRL1::LUC* reporter assays. *35S::RLUC* was used as an internal control. Experiments were repeated three times with similar results. Asterisks indicate significant differences from WT (**P < 0.05 and ***p < 0.001, Student's t‐test). D) Activation of the *pSlSBRL1: GUS* in hypocotyls at different time points with or without melatonin treatment in WT and *slpmtr1‐1*;*slpmtr2‐91*. E) Hypocotyl cuttings from 10‐day‐old tomato seedlings were treated with or without 10 µm melatonin, and photographs were taken after 6 days. F) AR numbers in different lines were quantified after 6 days of treatment with or without 10 µm melatonin. The data are presented as means ± SD from at least three independent experiments (n≥40 cuttings). Different letters indicate significant differences (*p* < 0.05, one‐way ANOVA, Tukey's test). G) Root primordia were stained 4 days after primary root removal from hypocotyls. H) Root primordia numbers were quantified after 4 days of treatment with 10 µm melatonin. The data are presented as means ± SD from at least three independent experiments (n≥10 cuttings), with different letters indicating significant differences (*p* < 0.05, one‐way ANOVA, Tukey's test).

Among these 715 genes coregulated by melatonin and SlPMTR1/2, *SlSBRL*/*SlLBD29* (*Solyc09g066270*) was reported as a crucial factor for AR induction, as its loss prevents ARs formation after the primary root is cut.^[^ ^8^ ^]^ This gene likely plays a role in phytomelatonin‐induced ARs through SlPMTR1/2 mediation. We detected the expression levels of *SlSBRL1* on the third day after applying with or without melatonin to WT and *slpmtrs* after cutting the primary root (**Figure** **6**A). Melatonin significantly increased the expression of *SlSBRL1* in WT, while it did not function in the *slpmtrs*. This indicates that melatonin activates the expression of *SlSBRL1*, a process dependent on the phytomelatonin receptor SlPMTR1/2. Within 96 h following injury, the expression level of *SlSBRL1* gradually increases, peaks at 9 h, and remains relatively high thereafter (Figure 6B). To further assess the role of SlPMTR1/2 in phytomelatonin‐SlSBRL1 signaling, we transiently expressed *SlPMTR1* or *SlPMTR2* in *slpmtr1‐1*;*slpmtr2‐91* protoplasts. The *slpmtr1‐1*;*slpmtr2‐91* mutant showed much lower *pSlSBRL1::LUC* expression than that in WT when treated with melatonin, and the expression of *SlPMTR1* or *SlPMTR2* restored the *pSlSBRL1::LUC* expression to near WT level (Figure 6C). These results indicate that phytomelatonin regulates the expression of *SlSBRL1* in a SlPMTR1/2 dependent manner.

2.6.1

*GUS* transgenic tomato seedlings revealed that *SlSBRL1* was expressed in stems and leaves, with minimal expression in the hypocotyl under normal conditions, but highly induced when injury occurs (Figure S6C, Supporting Information). 6 h after excising the primary root, *SlSBRL1* was initially expressed in the pericycle, with its expression increasing and spreading over time (Figure S6D, Supporting Information). We transferred *pSlSBRL1::GUS* into *slpmtr1‐1*;*slpmtr2‐91* and investigated the response of *SlSBRL1* to melatonin treatment. GUS expression was observed at wounding sites of both *pSlSBRL1::GUS* and *slpmtr1‐1*;*slpmtr2‐91*;*pSlSBRL1::GUS* after wounding for 6 and 12 h. Melatonin treatment enhances GUS expression in *pSlSBRL1::GUS*, but not in *slpmtr1‐1*;*slpmtr2‐91*;*pSlSBRL1::GUS*. These results indicate that *SlSBRL1* expression responds to mechanical injury and is further enhanced by melatonin, which requires SlPMTR1/2 (Figure 6D). To further investigate the critical role of SlSBRL1 in phytomelatonin promoted ARs regeneration, we used the phytomelatonin‐responsive‐promoter‐*pSlPMTR2* to drive *SlSBRL1* expression in the *slpmtr1‐1*;*slpmtr2‐91* mutant and checked AR induction (Figure S6A,B, Supporting Information). Following melatonin treatment, the transformation of *pPMTR2::SlSBRL1* restored the ARs regeneration in *slpmtr1‐1*;*slpmtr2‐91* mutant (Figure 6E,F). The staining of root primordia also showed a similar increase after melatonin treatment (Figure 6G,H). These findings support that phytomelatonin‐SlPMTR1/2 signaling promotes ARs regeneration through activating *SlSBRL1* expression.

SlSBRL1, as a newly discovered transcription factor involved in tomato AR induction, its mechanism of action is still unclear. We observed genes related to cell wall development and cell enlargement among those co‐regulated by melatonin and SlPMTR1/2, suggesting they may be downstream targets of SlSBRL1. The LUC/REN test results show that SlSBRL1 can upregulate the expression of *SlGDSL, SlCell2, and SlEXP11* (Figure S7A, Supporting Information), confirming its role in phytomelatonin receptor‐dependent AR induction, and its ability to regulate phytomelatonin‐regulated genes to promote AR induction.

### 2.7 SlSBRL1 Mediates the Amplification of the Phytomelatonin Signal in Wound‐Induced ARs Regeneration

**Figure 7:**
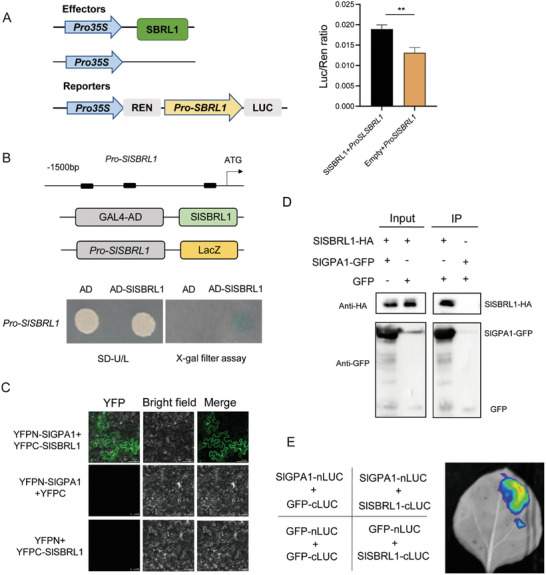
SlSBRL1 interacts with SlGPA1 and transcriptionally activates itself. A) Dual‐luciferase reporter assays show that SlSBRL1 activates its expression. The LUC/REN ratio represents the relative activity of the *SlSBRL1* promoter. Data represent mean ± SD of three independent replicates. Asterisks indicate a significant difference from the control according to the Student's t‐test at **p < 0.01. B) Schematic of the *SlSBRL1* promoter showing the G‐box (black box) and Yeast one‐hybrid assays confirmed SlSBRL1 binding to its promoter region. C) BiFC analysis of the interaction between SlGPA1 and SlSBRL1. Merge, merge of YFP and bright field images. D) Co‐immunoprecipitation assay showing that SlGPA1 interacts with SlSBRL1. SlGPA1‐GFP and SlSBRL1‐HA fusions were co‐expressed in *Nicotiana benthamiana* leaves. Proteins were extracted (Input) and immunoprecipitated (IP) with GFP agarose beads. The immunoblot assays were performed using anti‐GFP and anti‐HA antibodies. E) LCI assay of interaction between SlGPA1 and SlSBRL1. Different areas of tobacco leaves were co‐infiltrated with different pair constructs. SlSBRL1 and SlGPA1 were fused with cLUC and nLUC.

As mentioned above, *SlSBRL1* is upregulated by melatonin at the transcriptional level and acts as a transcription factor to regulate melatonin‐regulated genes. The results of the Y1H and LUC/REN assay indicate that SlSBRL1 binds to its promoter, enhancing its expression (**Figure** **7**A,B). SlSBRL1 is localized to both the cell membrane and the nucleus (Figure S7B, Supporting Information). Therefore, we are interested in how SlSBRL1 perceived the upstream phytomelatonin signal.

Upon phytomelatonin is perceived by the receptor AtPMTR1, AtGPA1 interacts with AtPMTR1 and transmits the signal to downstream effectors.^[^ ^26^, ^58^, ^59^ ^]^ We first identified the protein SlGPA1 (tomato G protein α subunit1) in tomatoes as the protein closely related to *Arabidopsis* GPA1 through homology analysis. The subcellular localization of SlGPA1 protein by transient overexpression of SlGPA1‐GFP in tomato protoplasts showed fluorescence overlapped with PIP2;1‐mCherry (Figure S8A, Supporting Information), indicating a membrane location. Immunoblotting detected the SlGPA1 signal in the total protein and membrane protein components (Figure S8B, Supporting Information). The *slgpa1* mutant showed no induction of adventitious root generation by melatonin, indicating SlGPA1's role in this process (Figure S8C–E, Supporting Information).

Through yeast‐based split‐ubiquitin system, Bi‐molecular fluorescence complementation (BiFC), luciferase complementation imaging (LCI), and protein pull‐down assay, we confirmed SlGPA1's interaction with the phytomelatonin receptor SlPMTR1/2 on the cytoplasmic membrane (Figure S9, Supporting Information) aligning with research findings in *Arabidopsis*.^[^ ^26^ ^]^ In plants, the interaction between multiple transmembrane proteins with G proteins generally occurs at the C‐terminal of the transmembrane protein.^[^ ^59^, ^60^, ^61^, ^62^ ^]^ SlPMTR1/2 was divided into N‐terminal and C‐terminal segments (SlPMTR1^△C^:1–134 aa, SlPMTR1^△N^:135–300 aa, SlPMTR2^△C^:1‐140 aa, SlPMTR2^△N^:141–309 aa) to investigate their interaction with SlGPA1. The results indicate that the interaction between SlPMTR1/2 and SlGPA1 occurs at the C‐terminal rather than the N‐terminal of SlPMTR1/2 (Figure S10, Supporting Information). The above results indicate that SlGPA1 interacts with the phytomelatonin receptor SlPMTR1/2 to facilitate MT signal transduction.

To study whether SlSBRL1 perceives the phytomelatonin signal from SlGPA1, we explored the interaction between SlSBRL1 and SlGPA1. BiFC assays showed that SlSBRL1 and SlGPA1 interact within the cell nucleus and cell membrane (Figure 7C). This was further confirmed by co‐immunoprecipitation (Co‐IP) and LCI assay (Figure 7D,E). These findings clarify the phytomelatonin‐regulated network involved in ARs generation via the SlPMTR1/2‐SlGPA1‐SlSBRL1 signaling cascade.

**Figure 8:**
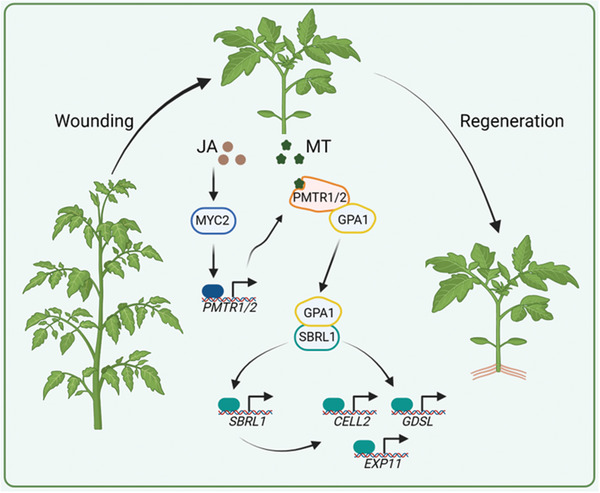
Phytomelatonin regulates adventitious root formation following mechanical injury. Melatonin applied to the wounded tomato stems is detected by SlPMTR1/2, which activates the downstream transcription factor SlSBRL1 through the G protein SlGPA1. SlSBRL1 amplifies the signal by enhancing its expression, promoting wound‐stimulated AR regeneration by regulating genes related to cell wall development and remodeling. Wounding also generates a large amount of JA hormone at the wound site, which activates SlMYC2. SlMYC2 promotes *SlPMTR1/2* expression, enhancing the phytomelatonin signal response and facilitating its interaction with JA in regulating AR formation.

Based on the above research, we propose a working model of phytomelatonin promoted wound‐inducible AR induction (**Figure** **8**). When melatonin was applied in the wounded seedlings, SlPMTR1/2 detects melatonin signals as a receptor and activates G protein SlGPA1, which then transmits signals to effector SlSBRL1. SlSBRL1 amplifies the signal by activating itself to further activate the expression of genes related to cell wall development and remodeling to accelerate the formation of AR. Meanwhile, JA activates regeneration pathways through its core transcription factor SlMYC2, which activates the expression of *SlPMTR1/2* to enhance phytomelatonin signal response and mediates synergistic effect with phytomelatonin in the wound‐inducible ARs regeneration.

## 3 Discussion

Adversity, or stress, comprises environmental factors detrimental to plant growth and survival. Due to their immobility, plants often endure stress from wounds caused by wind, rain, insects, animals, human activity, or machinery, relying on self‐healing mechanisms.^[^ ^1^, ^2^, ^3^, ^4^, ^63^ ^]^ When a branch is cut from the mother plant, it regenerates ARs at the wounding site, which is a commonly used plant cultivation and propagation method. Successful ARs induction is vital for the survival of the cuttings.^[^ ^6^, ^7^ ^]^ Studying ARs formation can help us better understand how plants cope with mechanical damage and can be applied to the large‐scale production of cloned plants in horticulture and forestry, improving production efficiency.

Previous studies have shown that phytomelatonin affects the transport and signal transduction of auxin, as well as the accumulation of auxin, through the NO signaling pathway to regulate the formation of ARs.^[^ ^10^, ^11^ ^]^ With the discovery of phytomelatonin receptors, it is unclear whether the effect of phytomelatonin on AR induction is achieved through its receptors. In this study, we confirmed the role of phytomelatonin in inducing ARs upon damage, as previously reported.^[^ ^10^, ^11^ ^]^ Root primordial staining,^[^ ^64^ ^]^ showed that melatonin treatment enhances root primordia development on the hypocotyl after wounding, leading to more ARs (Figure 1). Due to the positive effect of melatonin on AR induction, we explored its mechanism of action. We identified SlPMTR1 (*Solyc01g098210*) and SlPMTR2 (*Solyc06g069490*) as phytomelatonin receptors in tomatoes. Using MST experiments,^[^ ^41^ ^]^ we demonstrated the specific binding of SlPMTR1 and SlPMTR2 to melatonin (Figure 2D). Experiments with *slpmtr1/2* mutants revealed that phytomelatonin's regulatory effect on ARs is receptor‐dependent, and phytomelatonin's function is abolished in receptor mutants (Figure 2F–I).

JA is the hormone that responds quickly to plant injury and regulates AR development,^[^ ^22^, ^65^ ^]^ with SlMYC2 as its core transcription factor.^[^ ^56^, ^66^ ^]^ Crosstalk between phytohormones is crucial for plants to regulate plant activities precisely. In previous studies, the crosstalk between phytomelatonin and JA was mainly through regulating the expression of JA synthesis genes to affect JA content.^[^ ^52^ ^]^ The specific molecular mechanism and signal transduction pathway are not clear. We found that SlMYC2 specifically targets and activates the transcription of *SlPMTR1* and *SlPMTR2* (Figure 3), suggesting JA may enhance phytomelatonin signaling through SlMYC2. The expression patterns of *SlMYC2* and *SlPMTR1/2* in tomato seedlings after wounding suggest that they mediate immediate and prolonged effects, respectively (Figure S3D, Supporting Information). Acting downstream of SlMYC2, SlPMTR1/2 facilitates JA‐phytomelatonin crosstalk (Figure 4A–D). JA, as a rapid response hormone to injury, regulates ARs formation by activating ERF115/109 through the core transcription factor SlMYC2,^[^ ^22^, ^67^ ^]^ and enhancing phytomelatonin signaling through the SlMYC2‐*SlPMTR1/2* network for synergistic regulation. Given the detrimental effects of prolonged JA signaling on plants,^[^ ^22^ ^]^ the JA‐stimulated melatonin signal may play a crucial role in the extended process of adventitious root regeneration, serving as a secondary and slower pathway for ARs formation following injury. Therefore, since the primary JA‐SlMYC2‐ERF115/109 pathway remains functional, the *slpmtr1‐1*;*slpmtr2‐91* mutant shows a slight but non‐significant ARs reduction in hypocotyl experiments six days post‐cutting (Figure 4A–D). Over time, phytomelatonin activity increases, as the *slpmtr1‐1*;*slpmtr2‐91* mutant showed a significant ARs reduction in lateral branch experiments ten days post‐cutting (Figure S4D,E, Supporting Information). In the *slmyc2* mutant, where JA signaling is blocked, AR induction is reduced. However, activated MT signaling through SPMTR1/2 expression partially offset the impaired JA signaling, enhancing AR induction.

Previous transcriptome studies mostly focused on differentially expressed genes before and after melatonin treatment,^[^ ^68^, ^69^ ^]^ with limited research on phytomelatonin as a receptor‐dependent signal. Using the phytomelatonin receptor *slpmtr1/2* mutant, our transcriptome analysis confirmed SlPMTR1/2′s vital role in the phytomelatonin signaling pathway and identified genes specifically responsive to the phytomelatonin signal, co‐regulated by melatonin and SlPMTR1/2 (Figure 5). Among the co‐regulated genes, we identified the transcription factor SlSBRL1/SlLBD29, known for inducing ARs in tomatoes. The *slsbrl1* mutant cannot form ARs following injury.^[^ ^8^ ^]^ The LBD transcription factor family members have been reported to function in AR induction.^[^ ^12^, ^13^, ^70^ ^]^ We found that melatonin treatment can increase the expression level of *SlSBRL1* in WT but not in *slpmtrs* mutants (Figure 6A), indicating *SlSBRL1* responds to phytomelatonin via a receptor‐dependent signaling pathway. Using Ma's method,^[^ ^71^ ^]^ we showed SlPMTR1/2′s crucial role in upregulating *SlSBRL1* with melatonin treatment using the *pSlSBRL1::LUC* system (Figure 6C). GUS staining revealed that *SlSBRL1* is expressed in leaves and stems, but not in the hypocotyl, where its expression rises at the wounding site and is enhanced by melatonin, and effect is absent in *slpmtr1‐1*;*slpmtr2‐91* (Figure 6G). Overexpressing *pSlPMTR2::SlSBRL1* in *slpmtr1‐1*;*slpmtr2‐91* mutants resulted in increased ARs post‐melatonin treatment, unlike mutants alone (Figure 6E–H). However, without melatonin treatment, overexpressing *pSlPMTR2::SlSBRL1* failed to restore the AR regeneration in *slpmtr1‐1*;*slpmtr2‐91*, likely because cutting alone does not induce sufficient SBRL1 through *SlPMTR2* promoter when MT signaling is impaired. These results highlight the important role of SlSBRL1 in phytomelatonin‐regulated wound‐inducible AR development.

SlSBRL1 can bind to its promoter and enhance its expression to amplify upstream signals (Figure 7A,B), indicating its role in phytomelatonin signal transduction at both transcriptional level and protein levels. How SlSBRL1 perceives the phytomelatonin signal is worth exploring. Currently, phytomelatonin signal transduction downstream pathways are primarily the G protein and MAPK signaling cascades.^[^ ^26^, ^58^, ^72^ ^]^ We identified and confirmed that the tomato G protein α subunit1, named SlGPA1, interacts with phytomelatonin receptor SlPMTR1/2 (Figure S9, Supporting Information), and further with SlSBRL1, transmitting the phytomelatonin signal from SlPMTR1/2 to SlSBRL1 (Figure 7C–E). SlSBRL1 then amplifies the signal, initiating wound‐inducible AR regeneration. The interaction region between SlPMTR1/2 and SlGPA1 also explains why the *slpmtr* mutants fail to respond to melatonin signals (Figure S10, Supporting Information). The prematurely truncated SlPMTR1/2 is unable to properly interact with SlGPA1 and transmit MT signals due to the missing C‐terminal.

However, there are still some unexplained issues in the research. It is still unclear how the receptor SlPMTR1/2 perceives melatonin molecules and their specific functional domains. The mechanism by which the membrane protein SlGPA1 translocated to the nucleus to interact with transcription factor SlSBRL1 remains unclear. The activation and nuclear entry of the atypical G protein α subunit XLG1 may explain this phenomenon,^[^ ^71^ ^]^ but the specific mechanism still needs to be studied. Second, since SlSBRL1 enhanced its expression to amplify signaling, there must be a feedback regulatory mechanism to reduce SlSBRL1 levels to prevent stress reactions, but this mechanism is yet to be understood.

## 4 Experimental Section

### Plant Materials and Culture Condition

All experiments were conducted using the tomato (*Solanum lycopersicum* L.) cultivar Micro‐Tom. Tomato seeds were sterilized in a 3% NaClO solution and seeded on half‐strength Murashige and Skoog (1/2 MS) medium. Primary roots of 10‐day‐old seedlings were cut and seedlings were then transferred to a hydroponic system, supplied with standard Hoagland nutrient solution and melatonin treatment.^[^ ^73^ ^]^ Growth conditions were set at 16 h: 8 h, 26 °C: 18 °C, light: dark. Counting the number of ARs after 6 days. Tobacco plants used for transient transformation were grown in the incubator under the same conditions as above.

### Tomato Transformations

The target site used for the CRISPR/Cas‐9‐mediated genome editing of *SlPMTR1*, *SlPMTR2*, *SlMYC2*, and *SlGPA1*were selected by CCTop (https://cctop.cos.uni‐heidelberg.de:8043/index.html)^[^ ^74^ ^]^ and the guide RNA (sgRNA) containing the selected target sites was cloned into the binary vector pBSE402.^[^ ^75^ ^]^ The primers used are listed in Table S1 (Supporting Information). The final binary vector pBSE402 was transformed into Agrobacterium strain GV3101 and then into tomato cultivar Micro‐Tom following the published Protocol.^[^ ^76^ ^]^

### Staining of Root Primordium

Staining of root primordium was processed according to Zhang.^[^ ^64^ ^]^ Ten‐day‐old hypocotyls were selected to excise primary roots and stained after 4 days, as the root primordium is well developed but not yet differentiated into adventitious roots. After sampling, fix the hypocotyls in a fixed solution (70% ethanol: glacial acetic acid: formaldehyde = 18: 1: 1) and vacuum for 5 min, then store them in 70% ethanol. Before staining, wash with gradient ethanol and stain overnight with Schiff's reagent which was purchased from Coolaber (Beijing, China). After staining, rinse thoroughly and store in 10% glycerol. Observe the staining under a 10× stereo microscope. The experiments were performed three times with similar results.

### RNA‐Seq and RT‐qPCR Analysis

Wounded hypocotyl of *slpmtr1‐1*;*slpmtr2‐91* and wild type treated with or without melatonin were collected as three biological replicates. Total RNA was extracted using the RNA‐easy Plant Kit (Vazyme, Nanjing, China), and sequencing was performed by Majorbio Bio‐pharm Technology Co., Ltd. (Shanghai, China) using the 300‐bp paired‐end protocol on the Illumina HiSeq. 4000 platform. The raw reads were mapped to the tomato genome (https://solgenomics.net/ftp/tomato_genome/assembly/build_3.00/). Gene expression levels were expressed as fragments per kilobase of exon model per million reads mapped (FPKM), and differentially expressed genes were identified with the R package DESeq. 2 with *p*‐adjust < 0.05 and |log2 (fold‐change)| ≥ 1 as the threshold. Gene Ontology (GO) term enrichment was analyzed with the Majorbio Cloud Platform (www.majorbio.com). The raw data of RNA‐seq had been uploaded to NCBI with SRA (Sequence Read Archive): PRJNA1155853.

For RT‐qPCR, total RNAs were extracted and reverse transcribed with a PrimeScript RT reagent Kit (Takara, Japan). RT‐qPCR reaction was performed on QuantStudio 6 system (Applied Biosystems, USA) with RealStar Fast SYBR qPCR Mix (GenStar, China). Actin2 and EF1α were used as the reference genes. The primers used for RT‐qPCR are listed in Table S1 (Supporting Information).

### Microscale Thermophoresis (MST) Experiment

The MST experiment was carried out to determine the binding activity of SlPMTR1/2 to melatonin in vitro. Briefly, the coding sequence of maltose binding protein (MBP)‐SlPMTR1/2‐green fluorescent protein (GFP) fusion protein was subcloned into the pMAL‐c2 vector, and the resulting plasmid was introduced into *E. coli* strain Rosetta (DE3) competent cells (Shanghai Weidi Biotechnology) for protein expression. After 12 h of protein induction by 0.3 mm isopropyl‐beta‐D‐thiogalactopyranoside at 28 °C, the 6×Histidine (His)‐tagged MBP‐SlPMTR1/2‐GFP protein was purified using nitrilotriacetic acid (NTA)‐Ni beads (GE Healthcare) according to manufacturer's instructions. The MST assays were performed using NanoTemper monolith NT.115 (NanoTemper Technologies). One mg/ml of His‐MBP‐SlPMTR1/2‐GFP protein was incubated with varying concentrations of melatonin in MST reaction buffer for 30 min at 22 °C, and then the experiments were carried out using 80% excitation power and 20% MST power, a laser on time of 30 s. The original fluorescence data was collected from thermophoresis signal via NTAnalysis from three independent experiments to calculate the K_d_ value.

Similarly, an MST experiment was used to test the in vitro binding activity of SlMYC2 protein to *SlPMTR1/2* promoter fragments. In short, MBP‐His‐SlMYC2‐GFP protein was incubated with *SlPMTR1/2* promoter fragments in MST reaction buffer for 30 min at 22 °C, and then the experiments were carried out using 30% excitation power and 20% MST power. The original fluorescence data was collected from thermophoresis signal via NTAnalysis from three independent experiments to calculate the K_d_ value. The primers used are listed in Table S1 (Supporting Information).

### Subcellular Localization

The CDS, excluding the stop codon of SlPMTR1/2, was cloned into a pSuper1300 vector to form a *35S*::SlPMTR1/2‐GFP fusion construct. The primers used are listed in Table S1 (Supporting Information). PIP2;1‐mCherry fusion construct was used as an intrinsic plasma membrane protein marker.^[^ ^77^ ^]^ The construct and the control vector were respectively transformed into tomato protoplasts. For the mCherry protein, the excitation wavelength was set at 558 nm and the emission wavelength at 563–632 nm; for the GFP, the excitation wavelength was set at 488 nm and the emission wavelength at 495–545 nm. A LEICA TCS SP8 confocal microscope (Leica Microsystems Inc., Buffalo Grove, NY, USA) was used to detect the fluorescence signal. The experiments were performed three times with similar results.

### Protein Extraction and Western Blot Analysis

Plant total protein was extracted from the leaves (0.1 g fresh leaves per sample). The leaves were ground in liquid nitrogen and homogenized with 100 µL extraction buffer (50 mm HEPES, pH 7.5, 150 mm KCl, 1 mm EDTA, 0.5% Triton X‐100, 1 mm DTT, and 1 × protease inhibitor cocktail [Roche Diagnostics GmbH, Mannheim, Germany]). After the samples had been incubated on ice for 30 min, they were centrifuged at 12000 × g at 4 °C for 15 min, and the supernatant was then transferred to a new centrifuge tube, to which 5 × sodium dodecyl sulfate (SDS) loading buffer was added. The solution was boiled for 5 min followed by 5 min of centrifugation at 12000 × g at 4 °C. The samples were subsequently subjected to western blot analysis. Western blot analysis was conducted as described in the Definitive Guide to Western Blot (Abcam, Cambridge, UK). All monoclonal primary antibodies and secondary antibodies used here were purchased from TransGen Biotech (Beijing, China).

### Yeast‐One‐Hybrid (Y1H)

For Y1H assays, SlMYC2 was cloned into the pGAD‐424 vector, and 400‐bp promoter fragments (containing the G‐box) of *SlPMTR1* and *SlPMTR2* were cloned into the pLacZi vector. The primers used are listed in Table S1 (Supporting Information). The two vectors were transformed into the yeast YM4271 Chemically Competent Cell. Transformants were grown on the selective media SD medium lacking tryptophan and leucine (SD–Ura/Leu) and selected normal growing bacterial plaques for X‐gal filter assay. The experiments were performed three times with similar results.

### Luciferase (LUC)/ Renillia Luciferase (REN) Assays

For LUC/REN assays, the 700‐bp promoter fragments of SlPMTR1 and SlPMTR2 were cloned into pGreenII 0800‐miniLUC as reporters, whereas SlMYC2 were cloned into pGreenII 62‐SK as effectors. The primers used are listed in Table S1 (Supporting Information). The paired reporter and effector were transiently co‐expressed in 4‐week‐old tobacco leaves using Agrobacterium‐mediated transformation. Firefly and Renilla luciferase activity assays were then measured following the Dual‐Luciferase Reporter Assay System (Promega) with the GLOMAX 20/20 reader. The ratio of LUC to REN was used to reflect the transcriptional level of the promoter. The experiments were performed three times with similar results.

### Bi‐Molecular Fluorescence Complementation (BiFC)

*SlSBRL1* and *SlGPA1* coding sequences were cloned into the C‐terminus yellow fluorescent protein (cYFP) and N‐terminus YFP (nYFP) vectors, respectively. The resulting plasmids were transformed into *Agrobacterium* strain GV3101. Cell suspensions harboring appropriate pairs of vectors were co‐infiltrated into *N. benthamiana* leaves by *Agrobacterium*‐mediated infiltration. Images were captured using a LEICA TCS SP8 confocal microscope (Leica Microsystems Inc., Buffalo Grove, NY, USA). The experiments were performed three times with similar results.

### Co‐Immunoprecipitation (Co‐IP) Assays

For Co‐IP assays, SlSBRL1‐HA and SlGPA1‐GFP constructs were co‐infected into 5‐week‐old tobacco leaves. After 3 days, the total protein was extracted using native lysis buffer as described previously. Immunoprecipitation was performed with GFP‐trap magnetic beads (Chromotek, Munich, Germany) and eluted with glycine‐elution buffer. Samples were analyzed by western blot using GFP and HA antibodies. The experiments were performed three times with similar results.

### Luciferase Complementation Imaging (LCI)

For LCI assays, SlGPA1‐nLUC, SlSBRL1‐cLUC, and SlPMTR1/2‐cLUC constructs were generated and infiltrated into different areas of 4‐week‐old tobacco leaves. LCI images were taken with a charge‐coupled device (CCD) camera. GFP‐nLUC and GFP‐cLUC were set as negative controls. The experiments were performed three times with similar results.

### Pull‐Down Assays

SlGPA1‐GST and SlPMTR1/2‐GFP constructs were generated and expressed in BL21 cells cultured overnight at 28 °C with 0.1 mm isopropyl β‐D‐1‐thiogalactopyranoside (IPTG). Proteins were then purified with GST/GFP agarose (Thermo Fisher, Waltham, MA, USA). SlGPA1‐GST was then incubated with SlPMTR1/2‐GFP in 5 ml of pull‐down binding buffer (20 mm Tris–HCl, pH 7.5, 150 mm NaCl, and 0.1% Nonidet P‐40) for 1 h at 4 °C. The mixture was pulled down with GST agarose beads and eluted with sodium dodecyl sulfate (SDS) loading buffer. Samples were analyzed by western blot with GST and GFP antibodies. The experiments were performed three times with similar results.

### Split‐Ubiquitin Assay

The yeast split‐ubiquitin system was used to investigate the interaction between SlPMTR1/2 and SlGPA1. Briefly, the full‐length SlPMTR1/2 and SlGPA1 were cloned into NubG and Cub vectors and co‐transformed into the NMY51 yeast strains. Protein–protein interaction was determined by X‐gal filter assay to detect β‐galactosidase activity and growth on minimal media lacking Leu, Trp, His, and Ade. The experiments were performed three times with similar results.

### Histochemical GUS Staining

For 10‐day‐old seedlings were treated with or without melatonin after cutting the primary root. The hypocotyl of cuttings was selected for GUS staining. GUS staining was carried out according to the instructions (Coolaber, China). Materials were fixed in 90% acetone for 20 min, then immersed in GUS staining solution and incubated overnight at 25—37 °C. The next day, materials were transferred to 70% ethanol and decolorized 2–3 times until the negative control turned white. Under a microscope, blue dots on the white background indicate the GUS expression sites.

### Statistical Analysis

Statistical analyses were performed using GraphPad software. Means were compared using One‐way ANOVA (*Tukey*‐test) or unpaired two‐tailed *t*‐test. The sample size in this study is mainly determined according to prior experiences, which are based on the reproducibility and statistical significance of the results during the experiments. The results of the statistical analyses are shown in the Source Data file.

### Accession Numbers

Sequence data from this article could be found in the Sol Genomics Network: SlPMTR1 (Solyc01g098210), SlPMTR2 (Solyc06g069490), SlMYC2 (Solyc08g076930), SlGPA1 (Solyc08g061220), SlSBRL1 (Solyc09g066270), SlEXP11 (Solyc04g081870), SlCELL2 (Solyc07g053540), SlGDSL (Solyc03g111550), SlACTIN (Solyc11g005330), SlEF1α (Solyc06g005060).

## Conflict of Interest

The authors declare no conflict of interest.

## Author Contributions

N.Z. and Y.G. performed conceptualization. Y.L. did methodology. Y.L., X.W., S.J., C.J., H.L., and C.L. performed investigation. Y.L. did visualization. Y.G. and N.Z. did supervision. Y.L. wrote the original draft. Y.L., N.Z., and Y.G. wrote the review and performed editing

## Supporting information



Supporting Information

Supplemental Table 1

Supporting Information

## Data Availability

The authors declare that the data supporting the findings of this study are available within the paper and its supplementary information files. All tomato genes involved in this study can be found at Sol Genomics Network (https://solgenomics.net/). Source data are provided in this paper. The raw data of RNAseq has been uploaded to NCBI with SRA (Sequence Read Archive): PRJNA1155853.
