# Supplementary material for: Integration of Phytomelatonin Signaling With Jasmonic Acid in Wound‐induced Adventitious Root Regeneration
Source: Adv Sci (Weinh). 2025 Jan 23;12(11):2413485. doi: 10.1002/advs.202413485 (PMC11923874; doi:10.1002/advs.202413485)
Supplement: Supplementary file 1 — Supporting Information [file ADVS-12-2413485-s002.pdf]

ADVANCED SCIENCE
Open Access

Supporting Information

for *Adv. Sci.*, DOI 10.1002/advs.202413485

Integration of Phytomelatonin Signaling With Jasmonic Acid in Wound-induced Adventitious Root Regeneration

Ying Liu, Xiaoyun Wang, Shirui Jing, Congyang Jia, Hongxin Li, Chonghua Li, Qiuyu He, Na Zhang and Yang-Dong Guo**

Supplementary Materials for

Integration of phytomelatonin signaling with jasmonic acid in wound-induced adventitious root regeneration

Ying Liu *et al.*

*Corresponding author. yaguo@cau.edu.cn (Y.-D. Guo)

This PDF file includes:

Figs. S1 to S9

Other Supplementary Materials for this manuscript include the following:

Supplemental Table S1
Source data

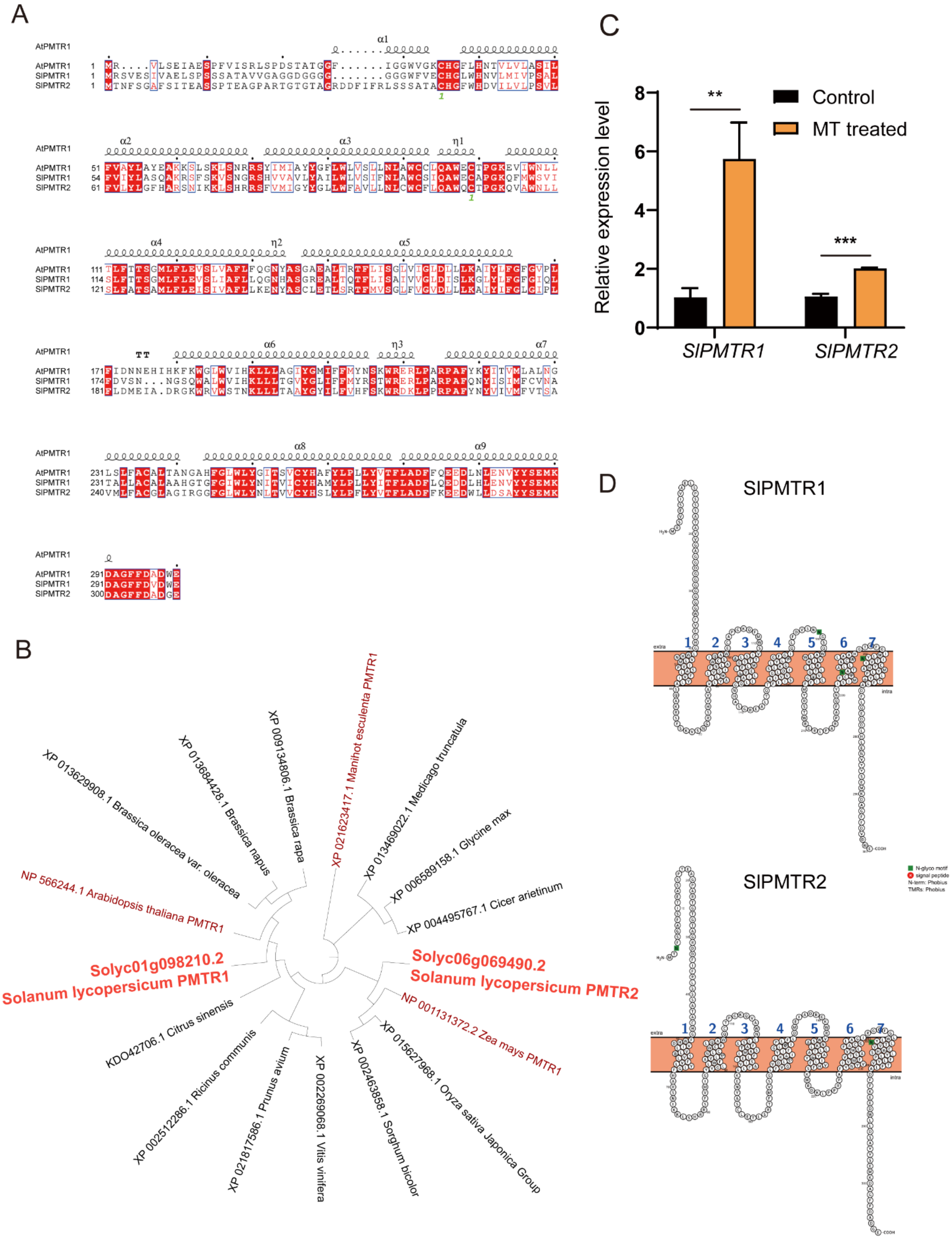
A
AtPMTR1
SlPMTR1
SlPMTR2
B
XP 013629908.1 Brassica oleracea var. oleracea
XP 013684428.1 Brassica napus
XP 009134806.1 Brassica rapa
XP 021623417.1 Manihot esculenta PMTR1
XP 013469022.1 Medicago truncatula
XP 006589158.1 Glycine max
XP 004495767.1 Cicer arietinum
NP 566244.1 Arabidopsis thaliana PMTR1
Solyc01g098210.2
Solanum lycopersicum PMTR1
Solyc06g069490.2
Solanum lycopersicum PMTR2
NP 001131372.2 Zea mays PMTR1
KDO42706.1 Citrus sinensis
XP 002512286.1 Ricinus communis
XP 021817586.1 Prunus avium
XP 002269068.1 Vitis vinifera
XP 002463858.1 Sorghum bicolor
XP 015627968.1 Oryza sativa Japonica Group
C
Relative expression level
Control
MT treated
**
***
SlPMTR1
SlPMTR2
D
SlPMTR1
SlPMTR2
extra
intra
N-glyco motif
signal peptide
N-term: Phobius
TMRs: Phobius

Fig. S1. Bioinformatics analysis of SlPMTR1/2. (**A**) Protein sequence alignment between *Arabidopsis* AtPMTR1 and the putative phytomelatonin receptor SlPMTR1/2 in tomato. The alignments were carried out using MEGA7 software. The identical amino acid residues are colored in red. The second structure was analyzed by ESPript 3.0 (https://espript.ibcp.fr/ESPript/cgi-bin/ESPript.cgi). The identical amino acid residues are colored with red. Labels starting by α and η refer to α-helices and 3_{10}-helices. Strict β-turns rendered as TT. Green digits at the bottom of sequences blocks show disulphide bridges. (**B**) Evolutionary relationships of AtPMTR1 were analyzed using the Neighbor-Joining method with MEGA7 program. (**C**) The expression of *SlPMTR1/2* in WT was examined with and without exogenous melatonin treatment. The data are presented as means ± SD from at least three independent experiments. Asterisks indicate significant difference from control according to Student's t test at $^{**}p < 0.05$ and $^{***}p < 0.001$. SlActin and SlEF1α were used as reference genes. (**D**) The predicted transmembrane structures of SlPMTR1/2 were analyzed using PROTEER (http://wlab.ethz.ch/protter/start/).

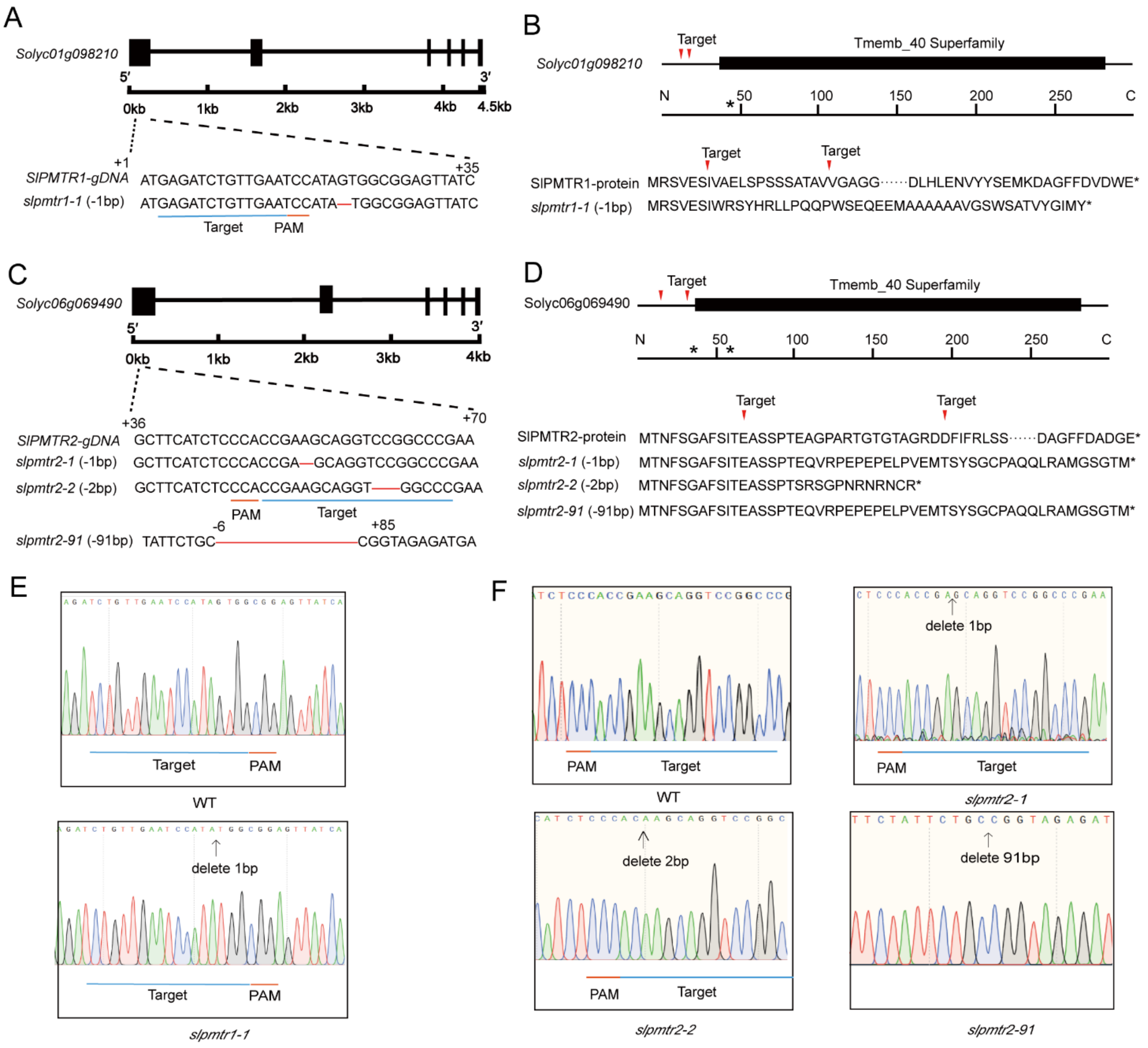

Fig. S2. The identification of CRISPR/Cas9 lines of *SlPMTR1/2*. (**A**) and (**B**) DNA and protein editing patterns of *slpmtr1-1*. (**C**) and (**D**) DNA and protein editing patterns of *slpmtr2-1*, *slpmtr2-2*, *slpmtr2-91*. * indicates the site of stop code. (**E**) The sequencing peak plots show the absence of bases of *slpmtr1-1*. (**F**) The sequencing peak plots show the absence of bases of *slpmtr2-1*, *slpmtr2-2* and *slpmtr2-91*.

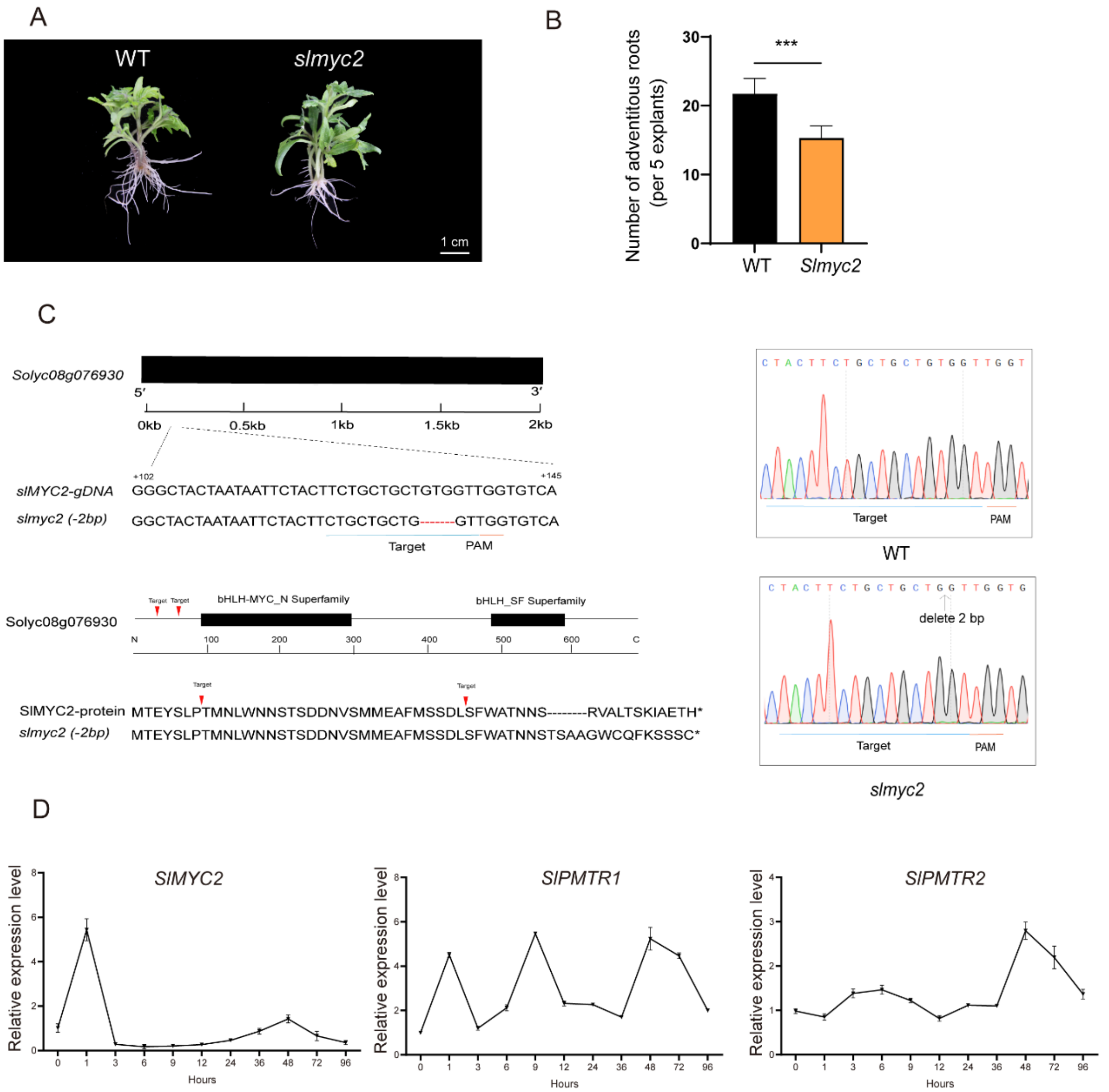

Fig. S3. The effects of SlMYC2 and JA on the induction of adventitious roots under mechanical injury. (**A**) The primary roots were cut from hypocotyls of 10-day-old germinated tomato seedlings. Photographs were taken after 6 days. (**B**) The number of ARs after 6 days of treatment. The data are presented as means ± SD from at least three independent experiments (n≥50 cuttings). Asterisks indicate significant difference from WT according to Student's t test at $^{***}p < 0.001$. (**C**) DNA and protein editing patterns of *slmyc2*. The sequencing peak plots show the absence of bases. **(D)** Temporal expression patterns of *SlMYC2*, *SlPMTR1* and *SlPMTR2* were analyzed in tomato seedling hypocotyls within 96 hours after the primary root was excised.

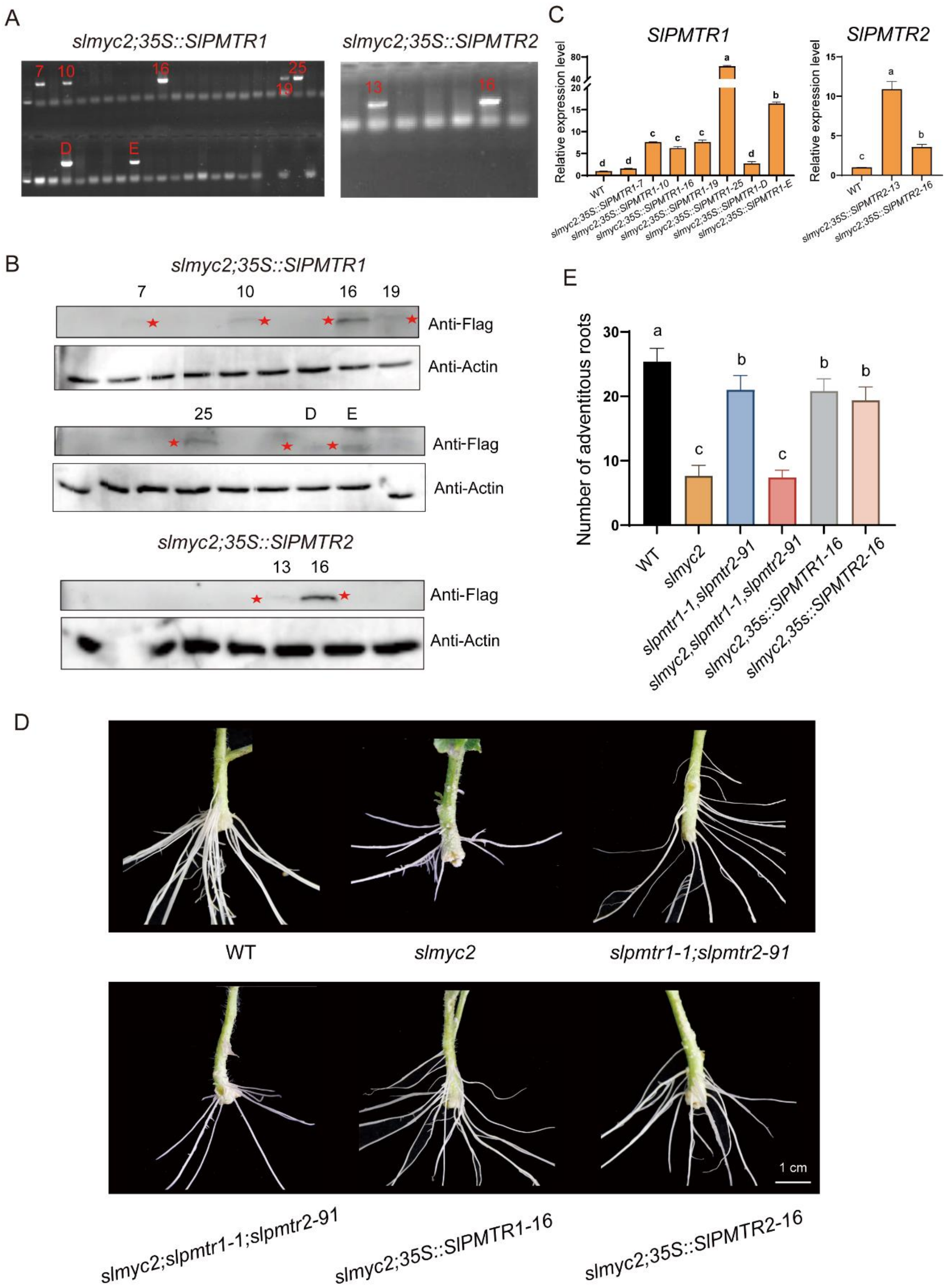

Fig. S4. Identification of overexpression materials and AR induction in lateral branches. (A) PCR analysis of the *SlPMTR1/2* overexpression lines. **(B)** Immunoblotting analysis showed that Flag antibodies recognize Flag labeled SlPMTR1/2. **(C)** The

expression of *SlPMTR1* and *SlPMTR2* in the transgenic lines. The data are presented as means ± SD from at least three independent experiments. Bars with different letters indicate significant differences ($p < 0.05$, one-way ANOVA, Tukey's test). The numbers on the lane represent the number of transgenic lines. (D) AR regeneration of lateral branches of various lines 10 days after the cut. (E) Statistical analysis of AR numbers in lateral branch experiments. AR numbers of lateral branches in various lines were assessed 10 days after the cut, with data shown as means ± SD from at least three independent experiments ($n \geq 5$). Different letters indicate significant differences ($p < 0.05$, one-way ANOVA, Tukey's test).

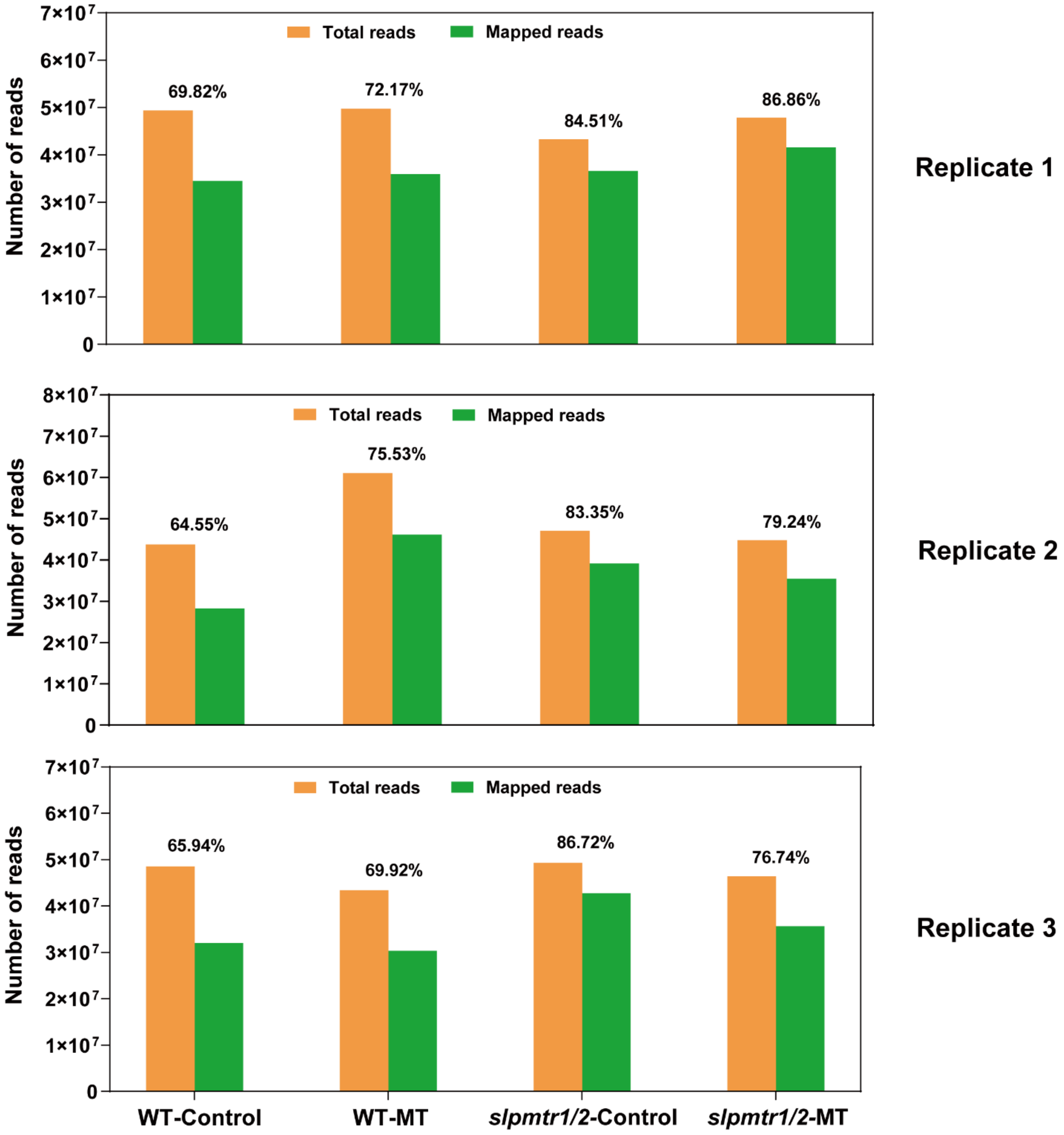

Fig. S5. Overview of RNA-seq data from melatonin-treated WT and *slpmtr1/2* mutants. Number of total reads, mapped reads and the percentage of mapped reads in each dataset are shown. This value was expressed in scientific notation.

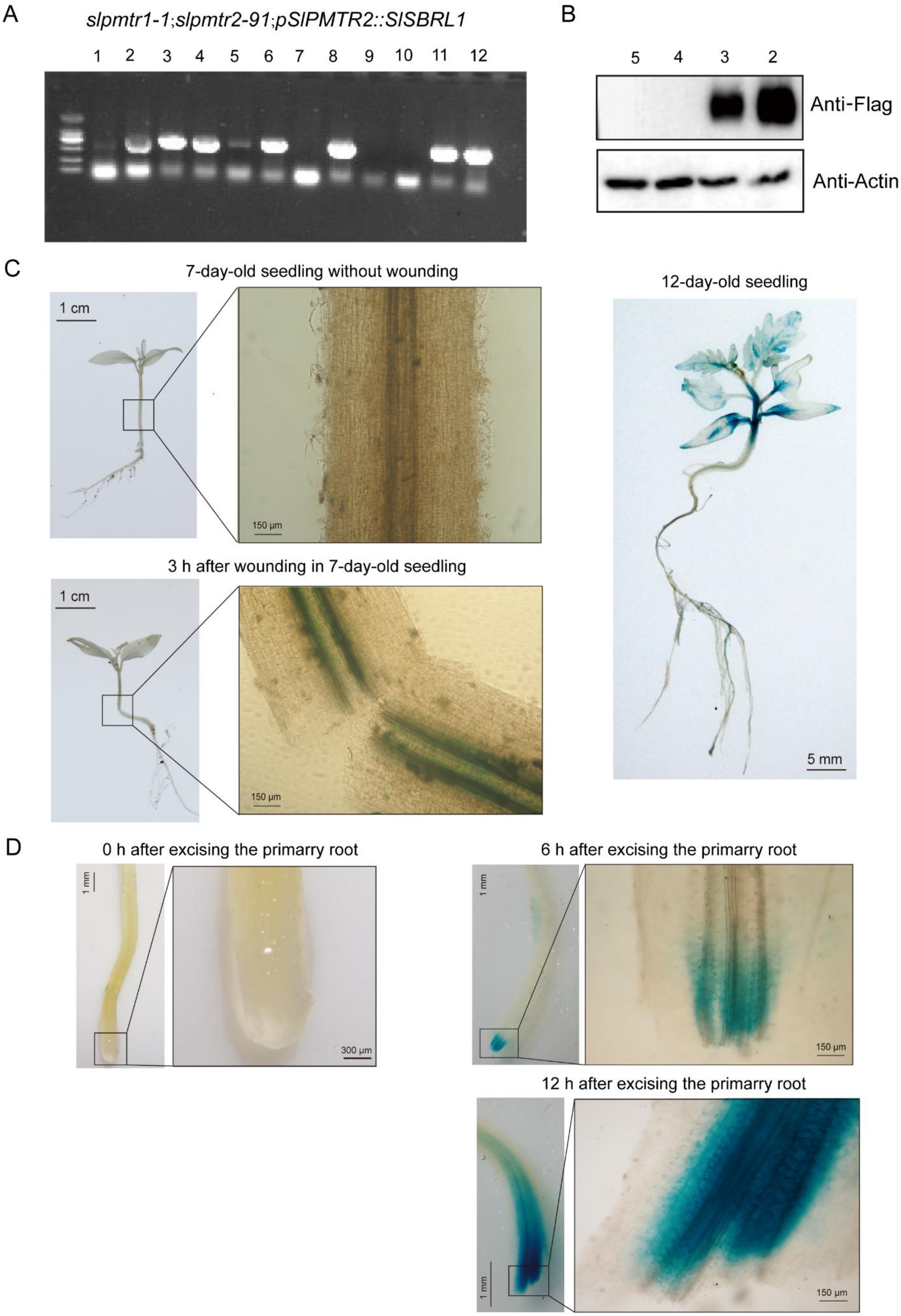

A
slpmtr1-1;slpmtr2-91;pSlPMTR2::SlSBRL1
1 2 3 4 5 6 7 8 9 10 11 12
B
5 4 3 2
Anti-Flag
Anti-Actin
C
7-day-old seedling without wounding
1 cm
150 μm
3 h after wounding in 7-day-old seedling
1 cm
150 μm
12-day-old seedling
5 mm
D
0 h after excising the primarry root
1 mm
300 μm
6 h after excising the primarry root
1 mm
150 μm
12 h after excising the primarry root
1 mm
150 μm

Fig. S6. ***SlSBRL1*** **is wound-induced.** (**A**) PCR analysis showed that *pSlPMTR2::SlSBRL1* was successfully transformed into *slpmtr1-1*;*slpmtr2-91*. The numbers on the lane represent the transgenic lines. (**B**) Immunoblotting analysis showed that Flag antibodies recognize Flag labeled SlSBRL1. The numbers on the lane represent the transgenic lines. (**C**) Expression patterns of the *pSlSBRL1::GUS* in tomato seedling. (**D**) Expression of *pSlSBRL1::GUS* in hypocotyl of 10-day-old seedling at different times after excising the primary root.

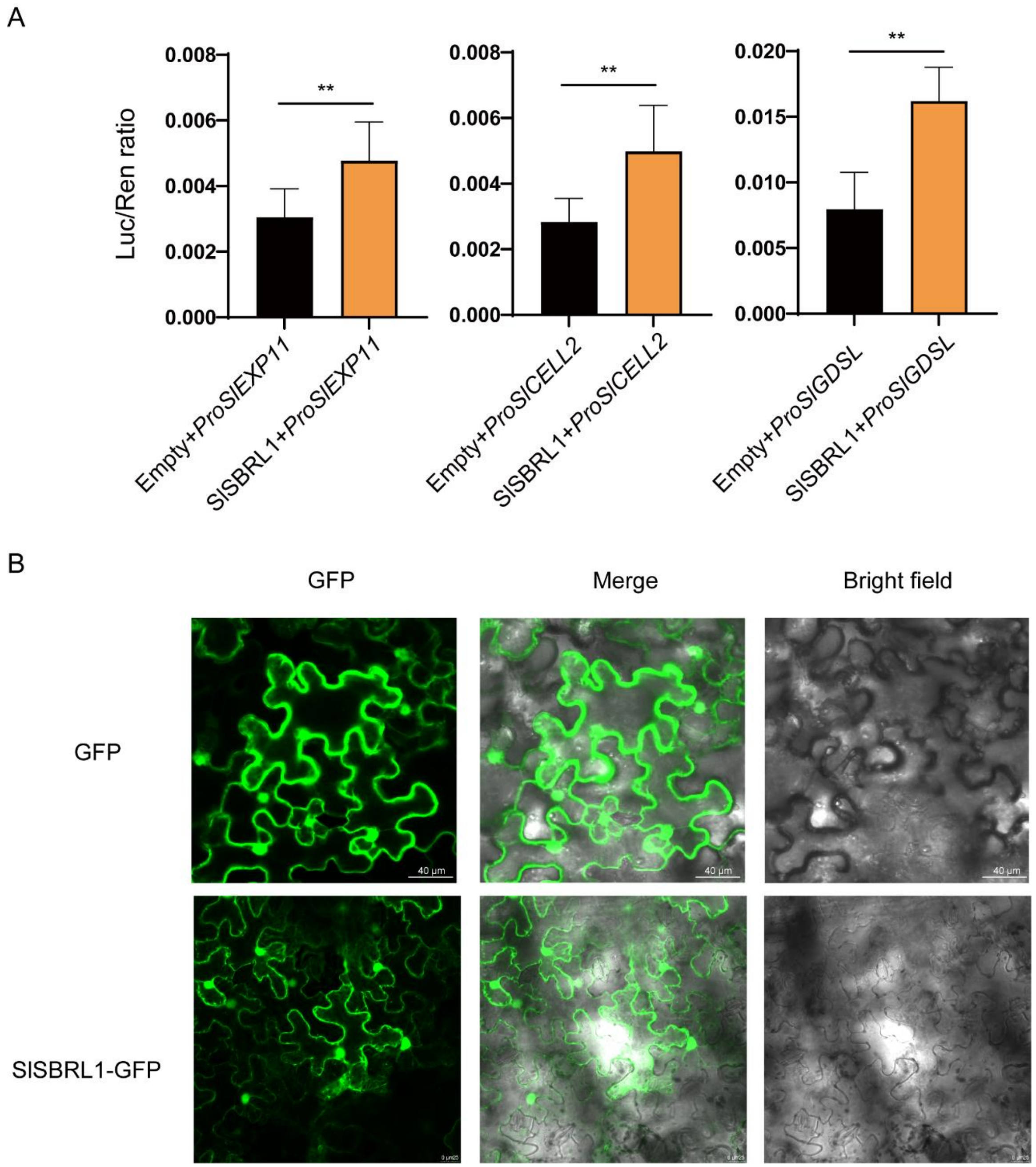

Fig. S7. SlSBRL1 regulates the expression genes related to cell wall development and binds to its own promoter. (**A**) Dual-luciferase reporter assays show that SlSBRL1 activates the expression of *SlGDSL, SlCell2, SlEXP11*. The LUC/REN ratio represents the relative activity of the *SlGDSL, SlCell2, SlEXP11* promoter. Data represent mean ± SD at least four independent replicates. Asterisks indicate significant difference from WT according to Student's t test at $**p < 0.01$. **(B)** Subcellular localization of SlSBRL1 in *Nicotiana benthamiana*. The GFP signals were visualized using confocal microscopy 48 h after transformation.

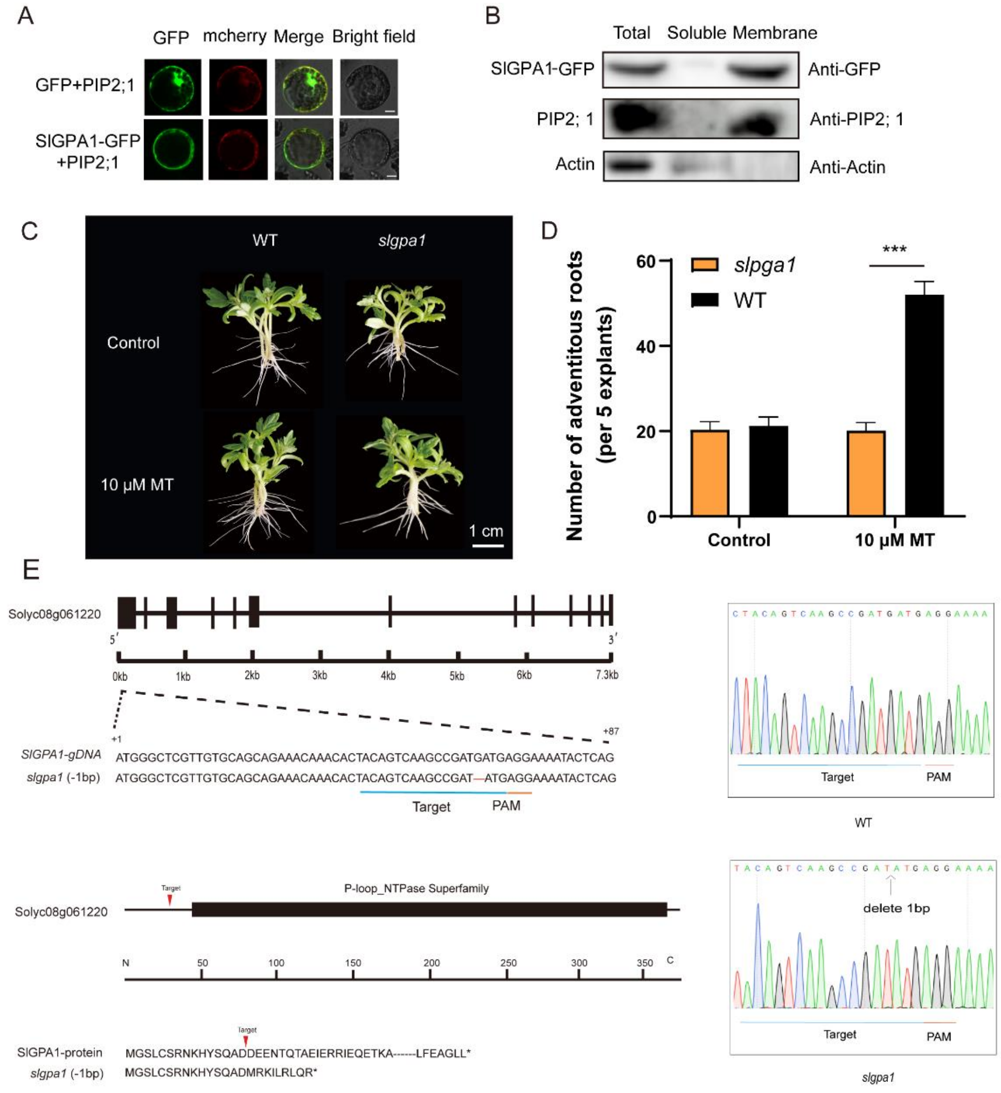

Fig. S8. Tomato SlGPA1 is a membrane protein involved in the regulation of melatonin on adventitious root regeneration. (**A**) Plasma membrane localization of SlGPA1 in tomato cells. SlGPA1-GFP signal was merged with PIP2;1-mCherry in tomato mesophyll protoplasts. The GFP and mCherry signals were visualized using confocal microscopy 48 h after protoplast transformation. Bar=25 µm. (**B**) Immunoblotting analysis showed that GFP antibodies recognize GFP labeled SlGPA1 in the membrane protein components of transgenic *Nicotiana benthamiana*. PIP2;1, membrane protein marker; Actin, soluble protein marker. **(C)** Primary roots from 10-day-old germinated tomato plants were cut and treated with or without 10 µM melatonin. Photographs were taken after 6 days of treatments. **(D)** The number of ARs in (C). The data are presented as means ± SD from at least three independent experiments (n≥45 cuttings). Asterisks indicate significant difference from WT according to Student's t test at ***$p < 0.001$. (**E**) DNA and protein editing patterns of *slgpa1*. The sequencing peak plots show the absence of bases.

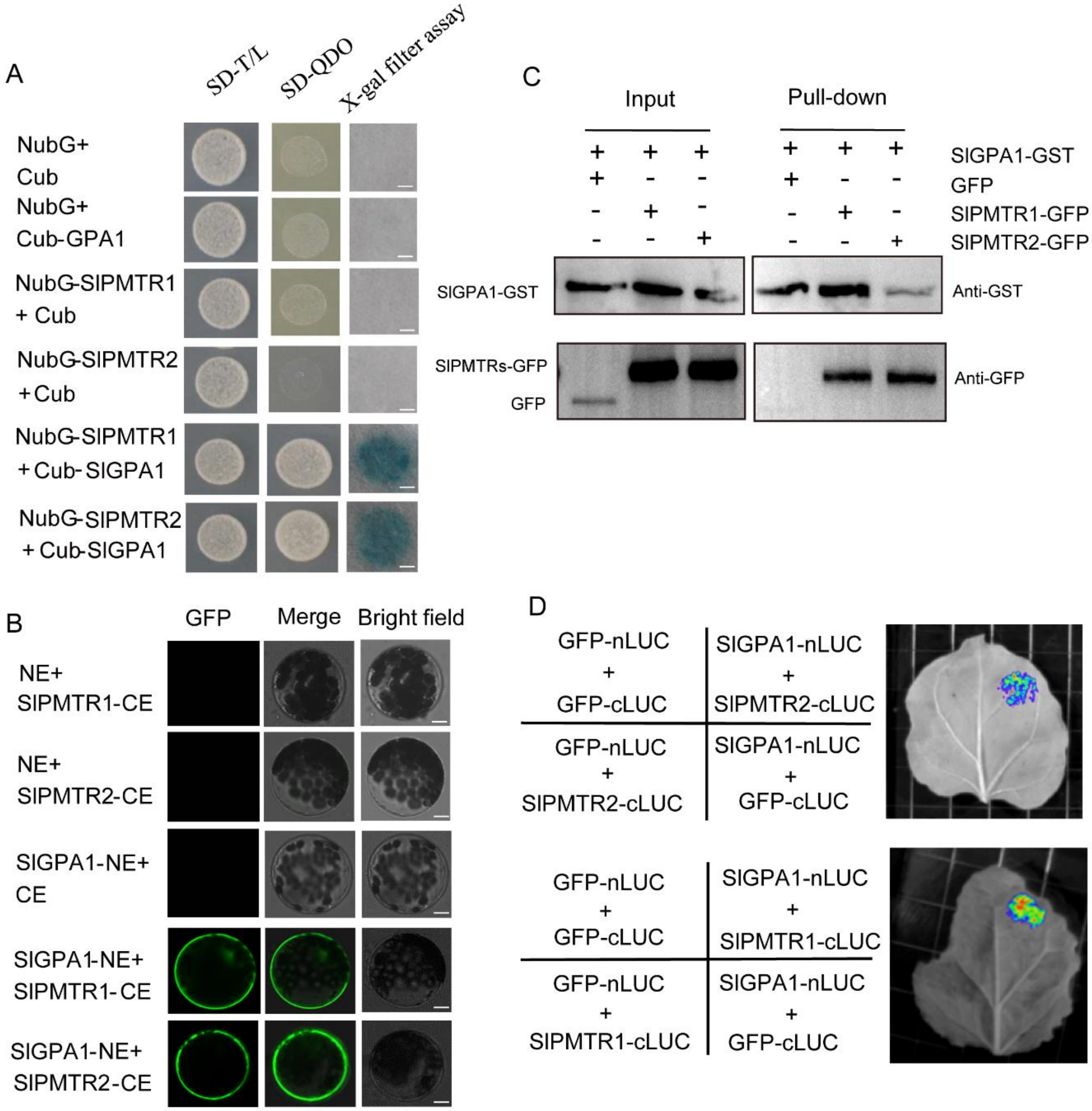

Fig. S9. SlGPA1 interacts with SlPMTR1/2. (**A**) SlPMTR1/2 and SlGPA1 interacted in the yeast-based split-ubiquitin system. The interaction between SlPMTR1/2 and SlGPA1 was determined by a growth assay on media lacking Leu (L), Trp (T), His (H), and Ade (A), by visualizing β-galactosidase activity using the X-gal overlay assay. Bar=200 mm. (**B**) BiFC analysis of the interaction between SlPMTR1/2 and SlGPA1. Merge, merge of YFP and bright field images. Bar=25 μm. (**C**) Pull-down assays show the interaction of SlPMTR1/2 with SlGPA1. GST-tagged SlGPA1 was incubated with amylose resin bound with SlPMTR1/2-GFP or GFP proteins, and the immune-precipitated proteins were detected with anti-GST antibody. (**D**) LCI assay of interaction between SlPMTR1/2 and SlGPA1. Different areas of tobacco leaves were co-infiltrated with different pair constructs. SlPMTR1/2 and SlGPA1 were fused with cLUC and nLUC.

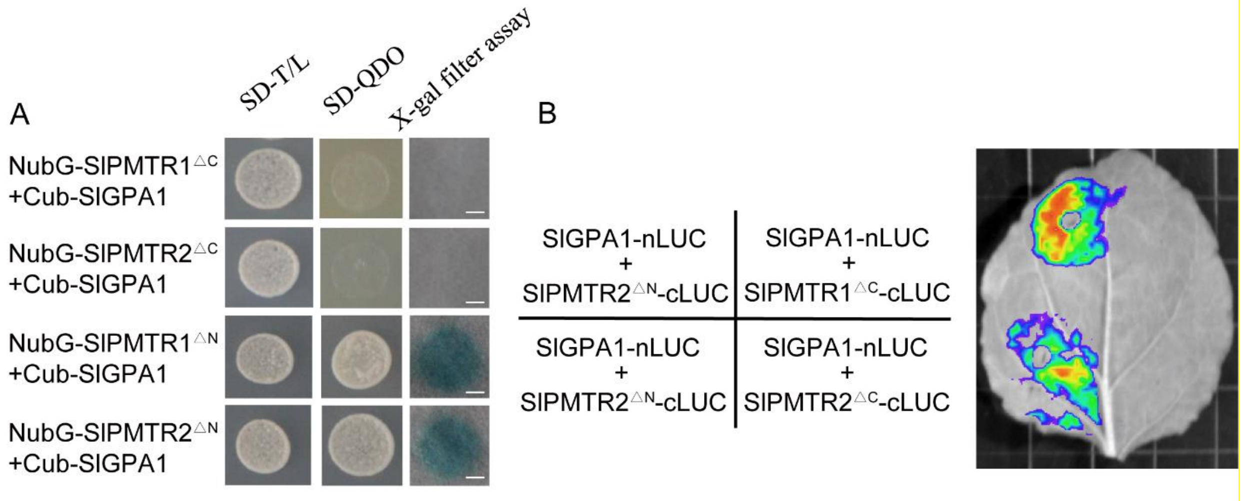

Fig. S10. SlGPA1 interacts with C-terminal of SlPMTR1/2. (**A**) The yeast-based split-ubiquitin system confirmed the interaction between the C-terminal region of SlPMTR1/2 and SlGPA1 through a growth assay on media lacking Leu (L), Trp (T), His (H), and Ade (A), and by visualizing β-galactosidase activity using the X-gal overlay assay. Bar=200 mm. (**B**) LCI assay verified the interaction by co-infiltrating various tobacco leaf areas with constructs where the C-terminal region SlPMTR1/2 and SlGPA1 were fused with cLUC and nLUC, respectively.
